# TXNRD1-driven enteric glial remodeling promotes neuronal dysfunction in Hirschsprung disease

**DOI:** 10.1016/j.isci.2026.117636

**Published:** 2026-10-01

**Authors:** Yanyi Li, Yuanli Wang, Zhu Jin, Chengyan Tang, Xinrong Xia, Yuan Gong, Qing Du, Lu Huang, Zeping Li, Yu Liao, Shengyin He, Bo Wang, Yuanmei Liu, Zebing Zheng

**Affiliations:** 1 Department of Pediatric Surgery, Children Hospital of Guizhou Province, Affiliated Hospital of Zunyi Medical University, Zunyi, Guizhou 563000, P.R. China

**Keywords:** Hirschsprung disease, enteric glial cells, TXNRD1, MAPK pathway, CXCL9:SPP1

## Abstract

Hirschsprung disease (HSCR) is characterized by distal intestinal aganglionosis and persistent dysfunction of the enteric nervous system after surgical treatment. Here, we integrated single-cell transcriptomics, network-based virtual perturbation analysis, proteomics, human tissue validation, enteric glial cell assays, neuron-glia co-culture systems, and an *Ednrb* conditional knockout (cKO) mouse model to investigate TXNRD1-dependent enteric glial remodeling in HSCR. We found that thioredoxin reductase 1 (TXNRD1) enrichment was associated with expansion of an SPP1-high/CXCL9-low enteric glial state and selective ERK/MAPK activation. TXNRD1 overexpression increased oxidative stress, impaired enteric glial migration and neurotrophic support, and induced neuronal dysfunction through suppression of GDNF/GFRα1/RET signaling. TXNRD1 inhibition alleviated HSCR-like phenotypes in *Ednrb* cKO mice. These findings identify TXNRD1-driven redox-dependent enteric glial remodeling as a mechanism contributing to neuronal dysfunction and HSCR progression.

## Highlights

• TXNRD1 is elevated in HSCR and enriches an SPP1-high enteric glial state
• TXNRD1-associated ROS activates ERK/MAPK signaling in enteric glial cells
• TXNRD1-impaired glia reduce neurotrophic support and disrupt neuronal function
• TXNRD1 knockdown improves HSCR-like phenotypes in *Ednrb* cKO mice

## Graphical abstract

**Figure:**
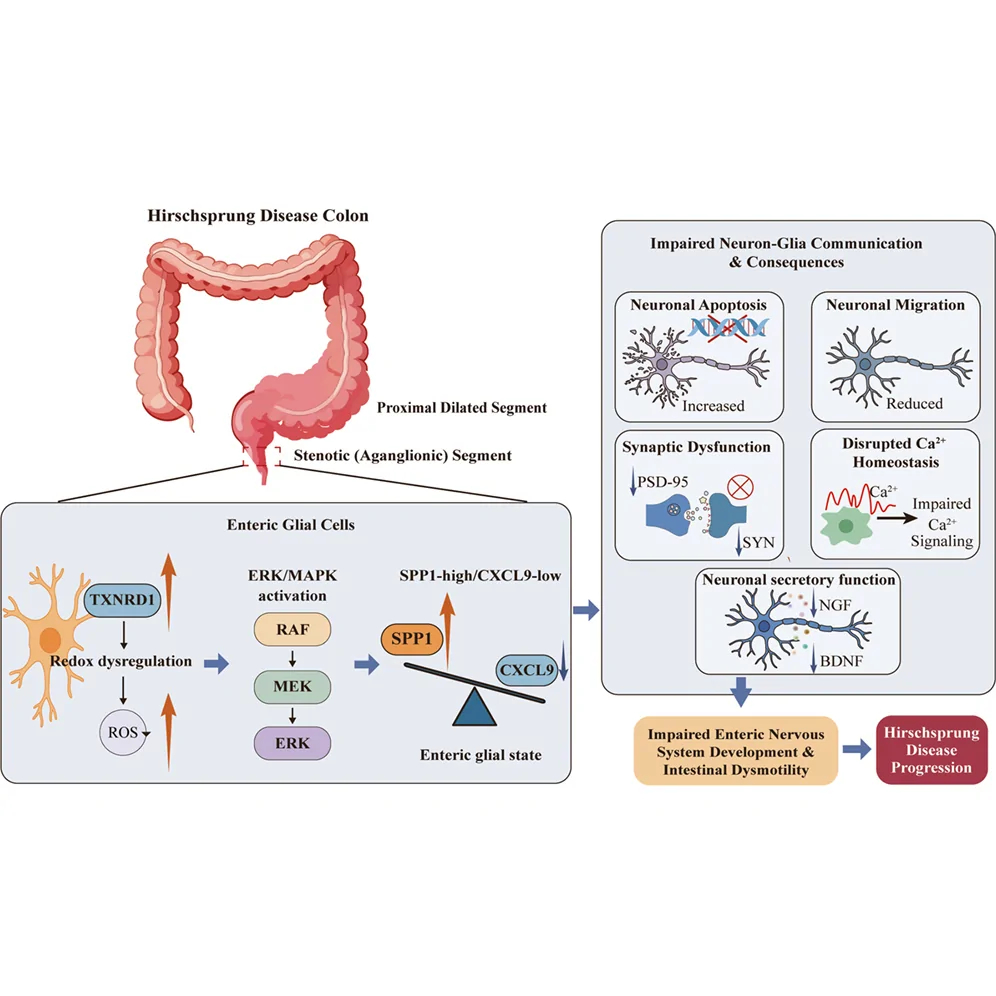

## Teaser

Cell biology; Developmental biology; Omics

## Introduction

Hirschsprung disease (HSCR) is a congenital disorder of the gastrointestinal tract resulting from failed migration and differentiation of enteric neural crest cells (ENCCs), leading to aganglionosis of the myenteric and submucosal plexuses in the distal intestine. Clinically, HSCR typically presents with abdominal distension and refractory constipation.^1^ Although surgical removal of the aganglionic segment followed by pull-through reconstruction remains the standard therapy,^2^^,^^3^ a substantial proportion of patients continues to experience long-term complications—including enterocolitis, anastomotic strictures, fecal soiling, persistent constipation, and the need for reoperation—which greatly diminish quality of life.^4^^,^^5^ These challenges underscore the urgent need to elucidate the molecular pathogenesis of HSCR and to identify potential therapeutic strategis.

Enteric glial cells (EGCs), the most abundant non-neuronal component of the enteric nervous system (ENS), play essential roles in maintaining epithelial integrity, coordinating neuro-immune signaling, and supporting neuronal survival.^6^^,^^7^ Recent single-cell RNA-sequencing (scRNA-seq) studies have identified a distinct SPP1^+^ glial subset with microglia-like features.^8^ In tumor-associated macrophages (TAMs), CXCL9 and SPP1 define mutually exclusive functional states associated with antitumor versus protumor activities—an axis termed CXCL9:SPP1 (CS) polarization. Moreover, a disease-associated SPP1^+^ microglial state has been implicated in ischemic brain injury, where Spp1-Cd44 signaling promotes inflammatory cell recruitment and aggravates tissue damage.^9^ Importantly, EGCs are recognized as a heterogeneous and plastic cell population, with single-cell and transcriptomic studies revealing dynamically regulated glial states during intestinal development, regeneration, and disease.^10^ Whether EGCs undergo a comparable CS-like polarization program during HSCR has not been established.

Thioredoxin reductase 1 (TXNRD1), a central regulator of intracellular redox homeostasis, governs thioredoxin-dependent redox cycling and modulates cellular proliferation, differentiation, and stress responses.^11^ Because redox imbalance can modulate ERK/MAPK signaling through reactive oxygen species (ROS)-sensitive mechanisms, aberrant TXNRD1 may contribute to disease progression by promoting redox-dependent remodeling of the MAPK pathway.^12^^,^^13^ However, whether TXNRD1 drives enteric glial state remodeling through redox-dependent ERK/MAPK signaling in HSCR has not been defined.

Here, we combined single-cell analysis, clinical validation, *in vitro* experiments, and an *Ednrb* conditional knockout (cKO) mouse model to define the role of TXNRD1 in HSCR. Network-based virtual perturbation analysis further supported TXNRD1 as an upstream regulator of redox- and MAPK-centered programs in EGCs. We show that TXNRD1 is upregulated in HSCR and is associated with the expansion of an SPP1-enriched EGC subcluster. Mechanistically, TXNRD1 promotes redox-dependent ERK/MAPK activation and drives EGCs toward an SPP1-high/CXCL9-low enteric glial state. This abnormal glial state impairs neurotrophic support and disrupts neuronal function, at least in part through suppression of GDNF/GFRα1/RET signaling. Together, these findings identify TXNRD1 as a regulator of pathogenic enteric glial remodeling in HSCR and provide a mechanistic framework linking redox dysregulation, glia-neuron interactions, and intestinal dysfunction.

## Results

### Single-cell analyses identify a TXNRD1-associated SPP1-enriched EGC subcluster and its regulatory features in HSCR

Integrated scRNA-seq analysis identified multiple cell populations in the enteric microenvironment, including neuro-glial, immune, and stromal-vascular compartments (Figures 1A and S1A). Given the established role of EGCs in HSCR, we further extracted the EGC compartment for secondary clustering and identified five transcriptionally distinct subclusters (Figures 1B and S1B). Among these, the C1 subcluster was characterized by the enriched expression of TXNRD1 and SPP1 (Figures 1C and 1D). Compared with normal controls, HSCR samples showed a marked expansion of C1-Spp1-EGCs, accompanied by a reduction in C5-Apoe-EGCs (Figure 1E).

**Figure 1:**
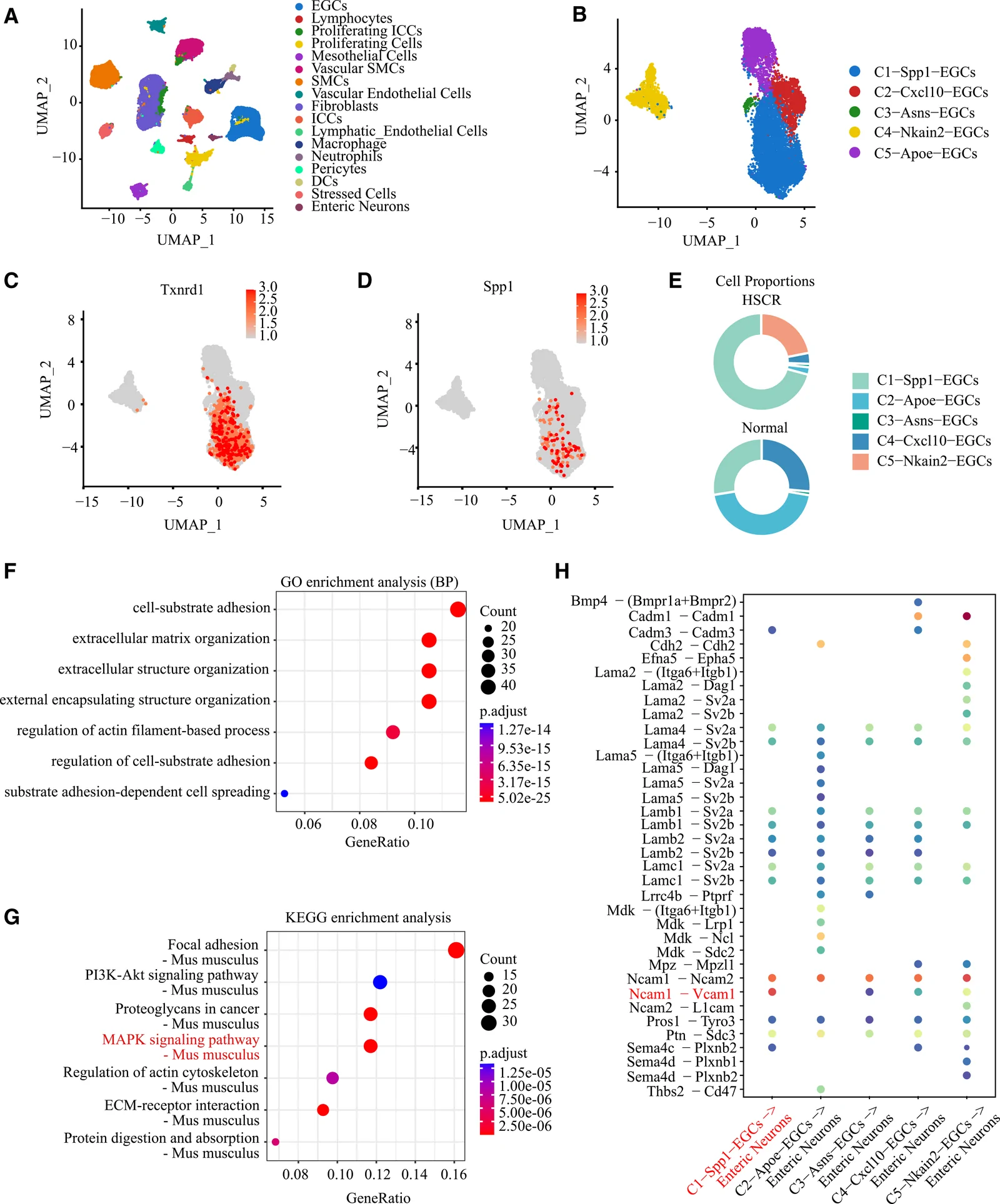
Single-cell transcriptomics identifies expansion of a *Txnrd1*-enriched *Spp1*-EGC subcluster and altered glia-neuron signaling in HSCR (A) Uniform manifold approximation and projection (UMAP) of the integrated scRNA-seq dataset showing the major neuro-glial, immune, epithelial, and stromal-vascular cell populations. (B) UMAP of re-clustered EGCs showing five transcriptionally distinct subclusters. (C and D) Feature plots showing the distributions of *Txnrd1* (C) and *Spp1* (D) transcripts across the EGC subclusters. (E) Donut plots showing the relative proportions of the five EGC subclusters in wild-type (WT) control and HSCR-model samples, including expansion of C1-Spp1-EGCs and reduction of C5-Apoe-EGCs in HSCR. (F) Biological-process enrichment analysis of C1-Spp1-EGC marker genes. (G) KEGG pathway enrichment analysis of C1-Spp1-EGC marker genes, highlighting focal-adhesion, PI3K-Akt, and MAPK-related programs. (H) CellChat bubble plot of predicted ligand-receptor interactions between EGC subclusters and enteric neurons, including the predicted *Vcam1*-*Ncam1* interaction. The publicly available scRNA-seq dataset GSE293138 comprised eight murine colonic tissue samples: proximal and distal colon from two WT mice and two *Ednrb*-null HSCR-model mice. The dataset comprised four tissue samples per genotype derived from two mice per genotype, with proximal and distal colon collected from each mouse. Individual cells are shown in (A)–(D). EGC subcluster proportions in (E) are descriptive summaries by genotype and were not subjected to inferential statistical testing. Enrichment *p* values were adjusted for multiple testing using the Benjamini-Hochberg method.

To explore the biological features of the expanded C1-Spp1-EGC subcluster, we performed Gene Ontology (GO) and Kyoto Encyclopedia of Genes and Genomes (KEGG) enrichment analyses on its marker genes. These analyses revealed enrichment in extracellular matrix organization, cell-substrate adhesion, actin filament regulation, focal adhesion, and the PI3K-Akt and MAPK signaling pathways (Figures 1F and 1G). In addition, CellChat analysis showed that, among all EGC subclusters, C1-Spp1-EGCs exhibited the highest number and strength of predicted interactions with enteric neurons (Figures S1C and S1D), and the ligand-receptor communication map highlighted a predicted VCAM1-NCAM1 interaction linking C1-Spp1-EGCs to enteric neurons (Figure 1H).

ScTenifoldNet-based virtual perturbation analysis further supported TXNRD1 as a regulator of enteric glial state remodeling. Both TXNRD1 virtual knockout (vKO) and virtual overexpression (vOE) converged on a shared regulatory axis involving redox control, ERK/MAPK-associated immediate-early response programs, and stress/reactivity-associated glial features. Under vKO, oxidative stress adaptation- and immediate-early response-related genes were prominently perturbed, with enrichment of MAPK, FoxO, HIF-1, TNF, NF-κB, PI3K-Akt, JAK-STAT, cytokine-cytokine receptor interaction, and ECM-receptor interaction pathways (Figures S2A and S2B). By contrast, vOE induced a more focused pathological shift, with SPP1 emerging as the most prominently perturbed gene, and pathway enrichment further supporting a pathological SPP1-centered EGC program linked to redox remodeling and ERK/MAPK activation (Figures S2C and S2D).

Together, these findings indicate that HSCR is associated with the expansion of a TXNRD1-enriched, SPP1-enriched EGC subcluster (C1-Spp1-EGCs), accompanied by altered glia-neuron communication and regulatory changes consistent with enteric glial state remodeling.

### TXNRD1 is upregulated in HSCR tissues and is associated with ERK/MAPK activation and an SPP1-high/CXCL9-low enteric glial state

Proteomic profiling of paired proximal dilated and stenotic colonic tissues from two children with HSCR revealed marked TXNRD1 upregulation in the stenotic segments (Figure 2A). Gene set enrichment analysis (GSEA) further demonstrated significant enrichment of the MAPK signaling pathway in HSCR tissues (Figures 2B and 2C), suggesting a potential association between TXNRD1 dysregulation and ERK/MAPK pathway activation.

**Figure 2:**
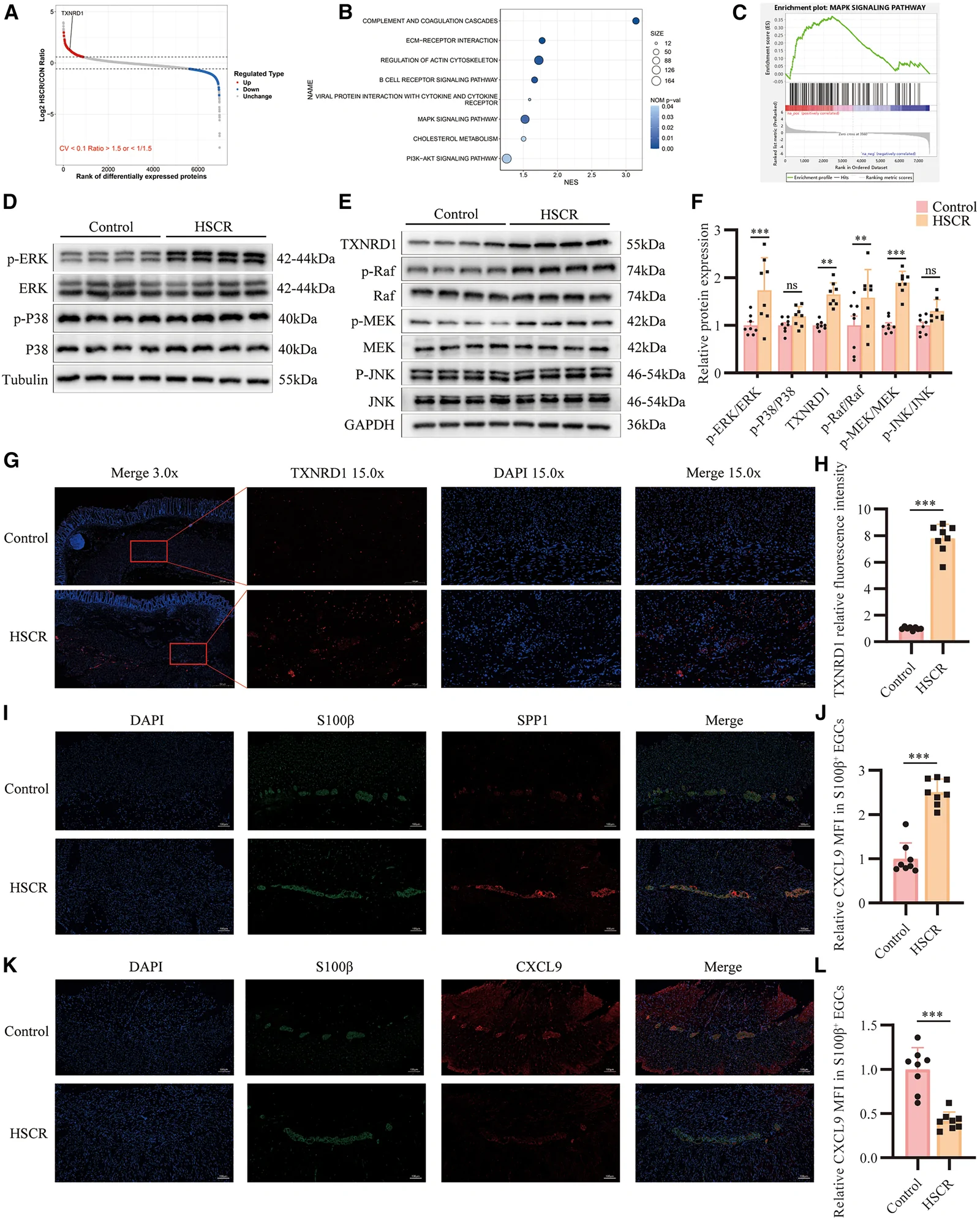
TXNRD1 upregulation in human HSCR tissues is accompanied by selective ERK/MAPK activation and an SPP1-high/CXCL9-low EGC state (A) Ranked distribution of differentially abundant proteins identified by liquid chromatography-tandem mass spectrometry (LC-MS/MS) proteomic analysis of paired proximal dilated and stenotic tissues; TXNRD1 is highlighted. (B) Gene set enrichment analysis (GSEA) of the proteomic dataset showing the principal enriched pathways. (C) GSEA enrichment plot for the MAPK signaling pathway. (D) Representative WB of p-ERK, ERK, p-p38, p38, and tubulin in paired tissues. (E) Representative WB of TXNRD1, p-Raf, Raf, p-MEK, MEK, p-JNK, JNK, and GAPDH in paired tissues. (F) Densitometric quantification of the protein ratios and TXNRD1 abundance shown in (D) and (E). (G and H) Representative IF images of TXNRD1 in paired colonic tissues (G) and quantification of relative TXNRD1 fluorescence intensity (H). (I and J) Representative IF images of SPP1 and the EGC marker S100β in paired colonic tissues (I) and quantification of SPP1 mean fluorescence intensity within S100β-positive EGCs (J). (K–L) Representative IF images of CXCL9 and S100β in paired colonic tissues and quantification of CXCL9 mean fluorescence intensity within S100β-positive EGCs. Paired proximal dilated and stenotic colonic tissues from children with HSCR were analyzed. The proteomic analysis included *n* = 2 paired patient samples. Tissue validation included *n* = 8 paired patient samples; each dot represents one patient, with proximal dilated and stenotic segments analyzed as a matched pair. Quantitative data are presented as mean ± SD with individual data points. Paired two-tailed Student’s t tests were used for (F), (H), (J), and (L). ∗∗*p* < 0.01, ∗∗∗*p* < 0.001; ns, not significant. Blots and micrographs are representative of the eight paired patient samples. Scale bars, 500 μm in the overview images and 10 μm in the magnified images in (G), and 100 μm in (I) and (K).

To validate these findings, paired stenotic and proximal dilated colonic tissues from HSCR patients were analyzed. Quantitative reverse-transcription PCR (RT-qPCR) and western blotting (WB) confirmed marked elevation of TXNRD1, accompanied by increased p-ERK/ERK, p-Raf/Raf, and p-MEK/MEK. In contrast, the ratios of p-p38/p38 and p-JNK/JNK showed no significant differences between groups (Figures S3A and 2D–2F). Immunofluorescence (IF) staining further verified enhanced TXNRD1 expression in stenotic segments compared with paired proximal dilated segments (Figures 2G and 2H).

Given the transcriptomic expansion of the SPP1-enriched EGC state in HSCR, we next examined whether TXNRD1 upregulation was accompanied by acquisition of a corresponding enteric glial state in human tissues. IF analysis showed that SPP1 expression was markedly increased in S100β^+^ EGCs in stenotic segments, whereas CXCL9 expression was reduced in the same cell population (Figures 2I–2L). Consistently, supplementary IF analysis confirmed increased SPP1 expression and reduced CXCL9 expression in stenotic segments compared with paired dilated tissues from HSCR patients (Figures S5C and S5D).

Together, these results show that TXNRD1 is significantly upregulated in HSCR tissues and is accompanied by selective ERK/MAPK activation and a shift of EGCs toward an SPP1-high/CXCL9-low enteric glial state.

### TXNRD1 drives a shift of EGCs toward an SPP1-high/CXCL9-low enteric glial state through ERK/MAPK activation

To determine whether TXNRD1 regulates EGC state remodeling through ERK/MAPK signaling, TXNRD1 was overexpressed in EGCs. This selectively increased ERK phosphorylation without activating JNK or p38 (Figures S3F and S3G), indicating pathway specificity. CCK-8 assay showed that SCH772984 at 10 nM had no significant effect on EGCs viability (Figure 3A).

**Figure 3:**
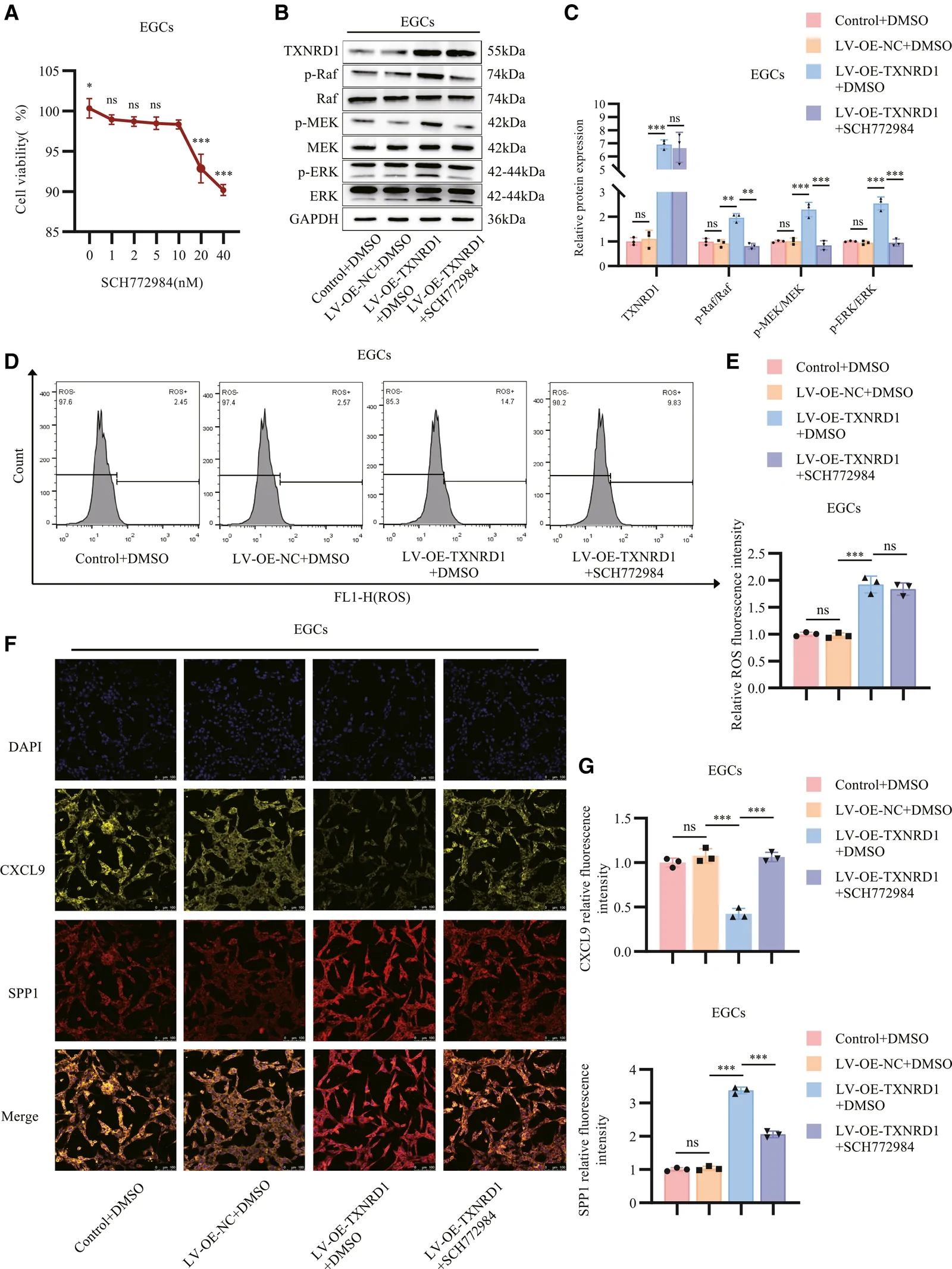
ERK inhibition attenuates TXNRD1-driven SPP1-high/CXCL9-low remodeling in EGCs (A) CCK-8 viability assay of cultured rat EGCs treated with the indicated concentrations of SCH772984 (0–40 nM) for 24 h; for (B)–(G), cultured EGCs were assigned to Control+DMSO, LV-OE-NC+DMSO, LV-OE-TXNRD1+DMSO, and LV-OE-TXNRD1+SCH772984 groups. SCH772984 was used at 10 nM. (B and C) Representative WB of TXNRD1, p-Raf, Raf, p-MEK, MEK, p-ERK, ERK, and GAPDH (B) and densitometric quantification (C). (D and E) Representative FCM histograms of intracellular ROS (D) and quantification of relative ROS fluorescence intensity (E). (F and G) Representative IF images of CXCL9 and SPP1 in cultured EGCs (F) and quantification of relative fluorescence intensity (G). *n* = 3 independent biological experiments; each dot represents one independently performed experiment. Quantitative data are presented as mean ± SD with individual data points. One-way ANOVA followed by Tukey’s multiple-comparison test was used. ∗*p* < 0.05, ∗∗*p* < 0.01, ∗∗∗*p* < 0.001; ns, not significant. Blots, FCM plots, and micrographs are representative of three independent experiments. Scale bars, 100 μm in (F).

To further test whether TXNRD1 promotes acquisition of an SPP1-high/CXCL9-low enteric glial state through ERK/MAPK signaling, cells were treated with the ERK inhibitor SCH772984 and assigned to four groups: Control+DMSO, LV-OE-NC+DMSO, LV-OE-TXNRD1+DMSO, and LV-OE-TXNRD1+SCH772984. WB analyses showed that TXNRD1 overexpression robustly increased p-ERK/ERK, p-Raf/Raf, and p-MEK/MEK levels, whereas SCH772984 effectively reversed these changes (Figures 3B and 3C).

IF analysis revealed that TXNRD1 overexpression significantly increased SPP1 expression and reduced CXCL9 expression, consistent with a shift toward an SPP1-high state. Notably, ERK inhibition partially restored CXCL9 expression and attenuated SPP1 upregulation (Figures 3F and 3G). Functionally, TXNRD1 overexpression impaired EGC migration and reduced secretion of GDNF and TNF-α, effects that were restored by ERK inhibition (Figures S3B–S3E). In addition, flow cytometry (FCM) showed that ROS levels were elevated after TXNRD1 overexpression, but were not significantly reduced by ERK inhibition (Figures 3D and 3E).

Together, these findings indicate that TXNRD1 drives EGC state remodeling toward an SPP1-high/CXCL9-low enteric glial state primarily through ERK/MAPK activation, while the associated redox dysregulation is not directly corrected by ERK blockade.

### TXNRD1-associated redox dysregulation contributes to ERK activation and promotes an SPP1-high/CXCL9-low enteric glial state

To further test whether TXNRD1-associated redox dysregulation contributes to ERK activation and acquisition of an SPP1-high/CXCL9-low enteric glial state, cells were treated with N-acetyl-L-cysteine (NAC), a widely used antioxidant and ROS scavenger. Cells were assigned to five groups: Control+DMSO, LV-OE-NC+DMSO, LV-OE-TXNRD1+DMSO, LV-OE-NC+NAC, and LV-OE-TXNRD1+NAC. WB analysis showed that TXNRD1 overexpression increased p-ERK/ERK levels, whereas NAC treatment attenuated ERK activation in TXNRD1-overexpressing EGCs (Figures 4A and 4B). FCM further demonstrated that intracellular ROS accumulation was elevated after TXNRD1 overexpression and was reduced by NAC treatment (Figures 4C and 4D). In parallel, TrxR activity was altered in TXNRD1-overexpressing cells and was partially restored after NAC intervention (Figure 4E).

**Figure 4:**
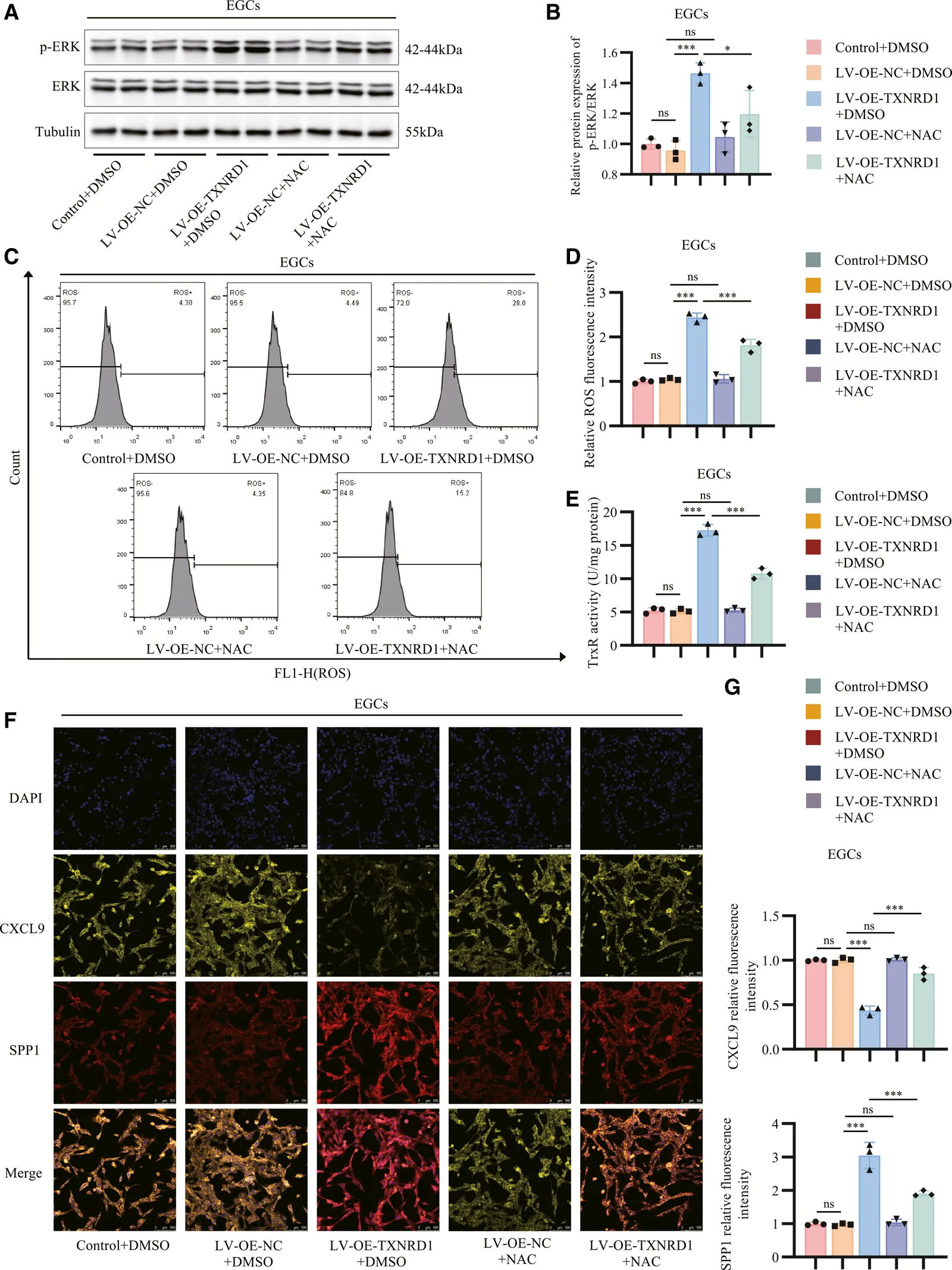
NAC suppresses TXNRD1-induced ROS accumulation, ERK activation, and SPP1-high/CXCL9-low remodeling in EGCs Cultured rat EGCs were assigned to Control+DMSO, LV-OE-NC+DMSO, LV-OE-TXNRD1+DMSO, LV-OE-NC+NAC, and LV-OE-TXNRD1+NAC groups. NAC was used at 1. (A and B) Representative WB of p-ERK, ERK, and tubulin (A) and quantification of the p-ERK/ERK ratio (B). (C and D) Representative FCM histograms of intracellular ROS (C) and quantification of relative ROS fluorescence intensity (D). (E) TrxR activity in EGC lysates. (F and G) Representative IF images of CXCL9 and SPP1 in cultured EGCs (F) and quantification of relative fluorescence intensity (G). *n* = 3 independent biological experiments; each dot represents one independently performed experiment. Quantitative data are presented as mean ± SD with individual data points. One-way ANOVA followed by Tukey’s multiple-comparison test was used. ∗*p* < 0.05, ∗∗∗*p* < 0.001; ns, not significant. Blots, FCM plots, and micrographs are representative of three independent experiments. Scale bars, 100 μm in (F).

IF analysis further showed that TXNRD1 overexpression markedly increased SPP1 expression and reduced CXCL9 expression, consistent with a shift toward an SPP1-high/CXCL9-low enteric glial state. Notably, NAC treatment partially reversed these changes, as evidenced by attenuation of SPP1 upregulation and restoration of CXCL9 expression in TXNRD1-overexpressing EGCs (Figures 4F and 4G).

Together, these findings indicate that TXNRD1-associated redox dysregulation contributes to ERK activation and promotes acquisition of an SPP1-high/CXCL9-low enteric glial state in EGCs.

To determine whether TXNRD1-overexpressing EGCs impair neuronal development and function through the GDNF/RET axis, primary dorsal root ganglia (DRGs) were co-cultured with EGCs from five experimental groups: Control+DMSO, LV-OE-NC+DMSO, LV-OE-TXNRD1+DMSO, LV-OE-TXNRD1+SCH772984, and LV-OE-TXNRD1+exogenous GDNF. FCM analysis showed that DRGs co-cultured with TXNRD1-overexpressing EGCs exhibited significantly increased apoptosis, whereas both ERK inhibition and exogenous GDNF partially attenuated this effect (Figures 5A and 5B).

**Figure 5:**
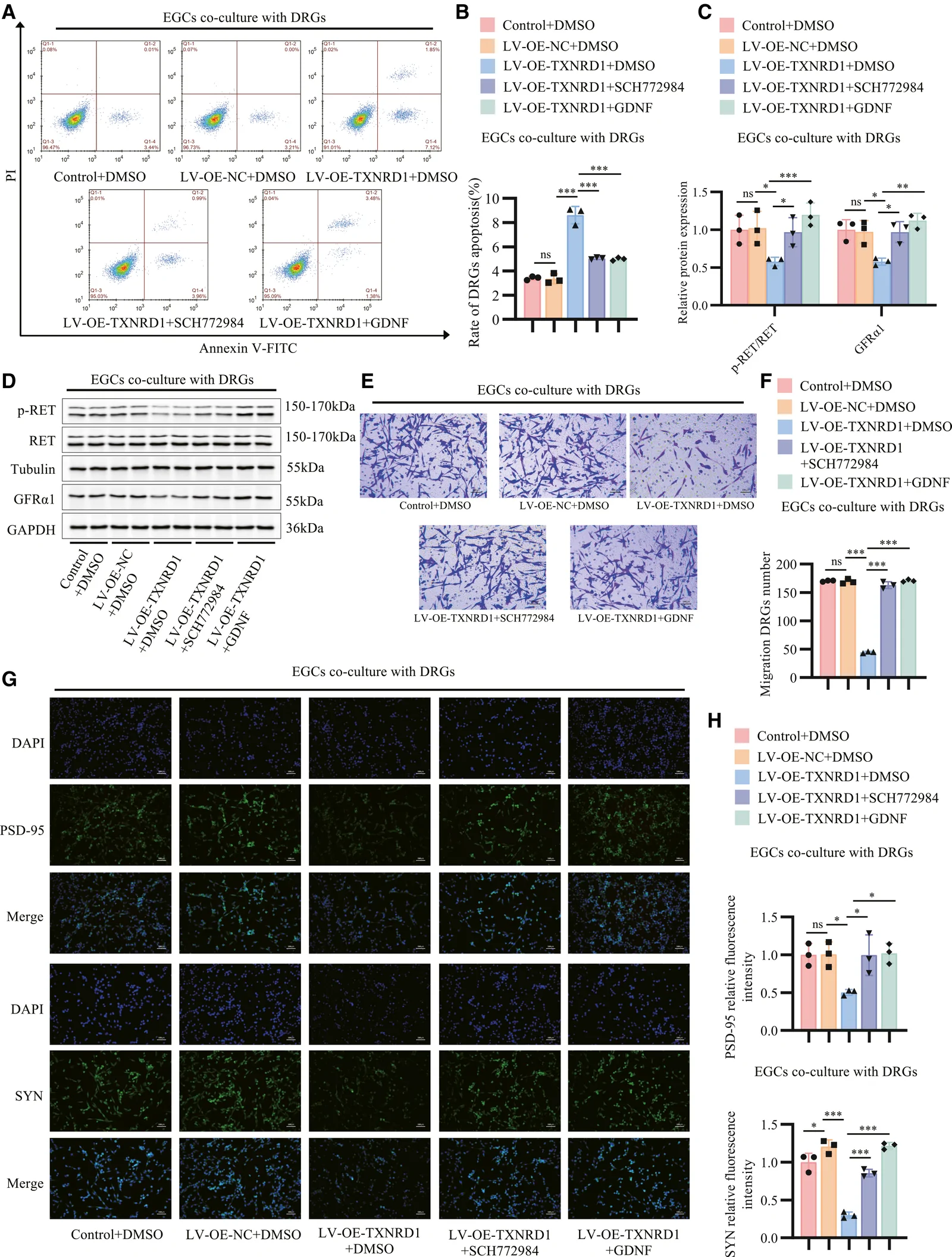
TXNRD1-overexpressing EGCs impair DRG neuronal function through suppression of GDNF/GFRα1/RET signaling Cultured rat EGCs were assigned to Control+DMSO, LV-OE-NC+DMSO, LV-OE-TXNRD1+DMSO, LV-OE-TXNRD1+SCH772984, and LV-OE-TXNRD1+recombinant GDNF groups and were co-cultured with primary rat dorsal root ganglion cells for 24 h. SCH772984 and GDNF were used at 10 nM and 50 ng/mL, respectively. (A and B) Representative Annexin V-FITC/propidium iodide FCM plots of DRG apoptosis (A) and quantification of the apoptotic-cell percentage (B). (C and D) Densitometric quantification of p-RET/RET and GFRα1/GAPDH (C) and representative WB of p-RET, RET, GFRα1, tubulin, and GAPDH in DRGs (D). (E and F) Representative crystal-violet images of migrated DRG cells (E) and quantification of migrated-cell number (F). (G and H) Representative IF images of the synaptic proteins PSD-95 and SYN in DRGs (G) and quantification of relative fluorescence intensity (H). *n* = 3 independent DRG-EGC co-culture experiments; each dot represents one independently performed co-culture experiment. Quantitative data are presented as mean ± SD with individual data points. One-way ANOVA followed by Tukey’s multiple-comparison test was used. ∗*p* < 0.05, ∗∗*p* < 0.01, ∗∗∗*p* < 0.001; ns, not significant. Blots, FCM, crystal-violet images, and micrographs are representative of three independent experiments. Scale bars, 100 μm in (E) and (G).

Crystal violet staining showed that TXNRD1-overexpressing EGCs markedly impaired neuronal migration, accompanied by decreased secretion of NGF and BDNF in the co-culture system. These impairments were partially reversed by SCH772984 or GDNF supplementation (Figures 5E, 5F, and S3H). WB analysis showed that co-culture with TXNRD1-overexpressing EGCs markedly reduced p-RET/RET and GFRα1 expression in DRGs, whereas both ERK inhibition and exogenous GDNF partially restored these changes (Figures 5C and 5D).

IF analysis further demonstrated that TXNRD1 overexpression downregulated the synaptic markers PSD-95 and SYN, while both interventions restored their expression (Figures 5G and 5H).

Moreover, FCM revealed abnormal intracellular Ca^2+^ accumulation in DRGs after co-culture with TXNRD1-overexpressing EGCs, which was partially alleviated after the interventions (Figures S4A and S4B).

Collectively, these findings indicate that TXNRD1-overexpressing EGCs impair neuronal development and function, at least in part, by suppressing the GDNF/GFRα1/RET signaling axis and weakening neurotrophic support in the glial-neuronal microenvironment.

### TXNRD1 promotes HSCR progression by inducing an SPP1-high/CXCL9-low enteric glial state in *Ednrb* cKO mice

To investigate the *in vivo* role of TXNRD1 in HSCR, *Ednrb* cKO mice were generated and administered adeno-associated virus (AAV)-sh-TXNRD1 by intraperitoneal injection or controls. Mice were divided into four groups: Control+PBS, cKO+PBS, cKO+AAV-sh-NC, and cKO+AAV-sh-TXNRD1. Efficient TXNRD1 knockdown was confirmed by RT-qPCR, WB, and IF in intestinal tissues, including S100β^+^ EGCs (Figures 6A, 6B, 6F, 6G, S4C, and S4E).

**Figure 6:**
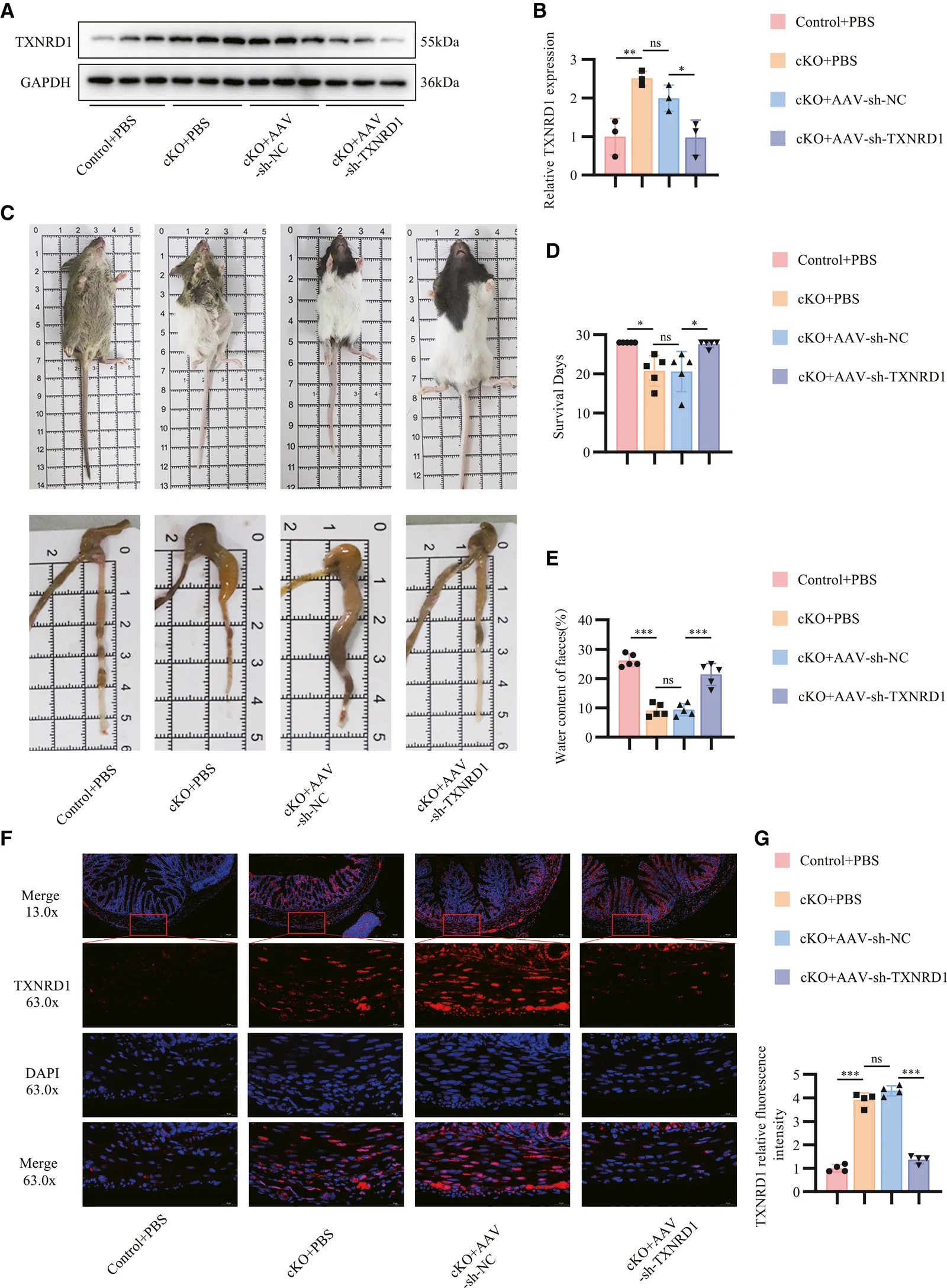
AAV-mediated TXNRD1 knockdown alleviates HSCR-like phenotypes in *Ednrb* cKO mice (A and B) Representative WB of TXNRD1 and GAPDH in intestinal tissues (A) and densitometric quantification of TXNRD1 abundance (B). (C) Representative external appearance and gross colonic morphology of mice in each group. (D) Survival duration to the humane or study endpoint. (E) Fecal water content. (F and G) Representative IF images of TXNRD1 in intestinal tissues (F) and quantification of relative TXNRD1 fluorescence intensity (G). Postnatal day 5 control and *Ednrb* cKO mice received intraperitoneal PBS, AAV-sh-NC, or AAV-sh-TXNRD1 as indicated. Each AAV-treated mouse received 2 × 10^11^ viral particles. For WB, *n* = 3 mice per group. For gross morphology and IF analysis, *n* = 4 mice per group. For survival and fecal-water-content analyses, *n* = 5 mice per group. Both male and female mice were included in the study. Each dot represents one mouse. Data in (B), (E), and (G) are presented as mean ± SD with individual data points and were analyzed by one-way ANOVA followed by Tukey’s multiple-comparison test. Survival distributions in (D) were compared using the log rank test. ∗*p* < 0.05, ∗∗*p* < 0.01, ∗∗∗*p* < 0.001; ns, not significant. Blots are representative of three mice per group, whereas gross photographs and micrographs are representative of four mice per group. Scale bars, 100 μm in the overview images and 20 μm in the magnified images in (F).

Phenotypically, TXNRD1 suppression markedly ameliorated HSCR-like features, including abdominal distension, proximal colonic dilatation, distal stenosis, and fecal retention (Figure 6C). Consistently, survival analysis showed that TXNRD1 knockdown significantly prolonged survival in cKO mice, with four animals surviving to the study endpoint (Figure 6D). Fecal water content was significantly increased after TXNRD1 knockdown and partially restored toward control levels, indicating an improvement in intestinal functional impairment (Figure 6E).

IF analysis further demonstrated that TXNRD1 knockdown reversed the abnormal enteric glial state *in vivo*, as evidenced by decreased SPP1 expression and restored CXCL9 expression in intestinal tissues of *Ednrb* cKO mice (Figures 7A and 7B). In parallel, the expression of the neuronal marker TUJ1 and the ENCC marker p75 was increased after TXNRD1 suppression (Figures 7C and 7D). Consistently, the cell-cell communication mediators NCAM1 and VCAM1 were also significantly upregulated (Figures S5A and S5B).

**Figure 7:**
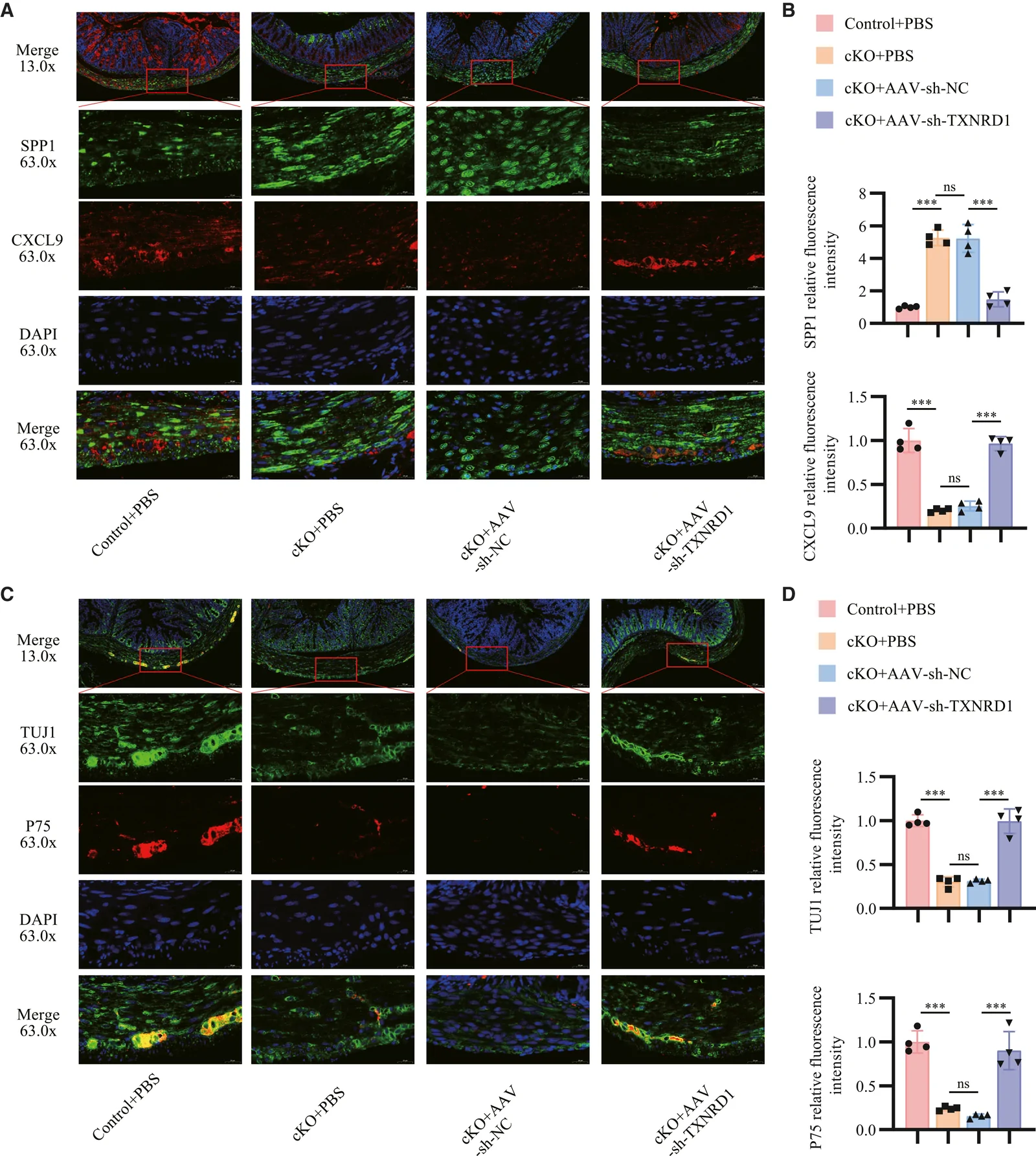
TXNRD1 promotes HSCR progression by inducing an SPP1-high/CXCL9-low enteric glial state in *Ednrb* cKO mice Mice were grouped identically to Figure 6. (A and B) Representative IF images of SPP1 and CXCL9 in intestinal tissues (A) and quantification of relative SPP1 and CXCL9 fluorescence intensity (B). (C and D) Representative IF images of the neuronal marker TUJ1 and enteric neural crest cell marker p75 in intestinal tissues (C) and quantification of relative TUJ1 and p75 fluorescence intensity (D). *n* = 4 mice per group; both male and female mice were included in the study, and each dot represents one mouse. Quantitative data are presented as mean ± SD with individual data points. One-way ANOVA followed by Tukey’s multiple-comparison test was used. ∗∗∗*p* < 0.001; ns, not significant. Micrographs are representative of four mice per group. Scale bars, 100 μm in the overview images and 20 μm in the magnified images in (A) and (C).

Collectively, these findings indicate that TXNRD1 promotes HSCR progression in *Ednrb* cKO mice by inducing an SPP1-high/CXCL9-low enteric glial state, thereby contributing to impaired neuronal integrity and intestinal dysfunction.

## Discussion

In this study, we identify TXNRD1 as a driver of an SPP1-high/CXCL9-low enteric glial state in HSCR. By integrating single-cell analysis, network-based virtual perturbation, human tissue validation, *in vitro* mechanistic experiments, and the *Ednrb* cKO model, we show that HSCR is associated with expansion of a TXNRD1-enriched, SPP1-enriched EGC subcluster. This subcluster displayed signaling features related to extracellular matrix organization, cell adhesion, focal adhesion, and PI3K-Akt/MAPK pathway activity, and exhibited the strongest predicted communication with enteric neurons, including a VCAM1-NCAM1 interaction axis. In parallel, virtual perturbation analysis supported TXNRD1 as a regulator of enteric glial state remodeling, with both vKO and vOE converging on redox- and ERK/MAPK-associated immediate-early regulatory programs, whereas vOE showed a stronger association with a pathological SPP1-centered program. Together, these findings support the view that EGCs in HSCR are not merely passive bystanders secondary to aganglionosis, but active participants in disease-associated enteric glial remodeling and glia-neuron microenvironmental dysfunction.

TXNRD1 is a core component of the thioredoxin-dependent antioxidant system.^14^^,^^15^ Experimental evidence has linked TXNRD1/TrxR1 dysregulation to ROS accumulation, MAPK activation, and pathological stress responses.^16^ Sang et al.^17^ showed that pharmacological targeting of TrxR1 induced ROS-mediated cell death with concomitant activation of MAPK-related signaling. In neuronal models, Liu et al.^18^ reported a protective role for TXNRD1 against oxidative stress and neuronal injury, indicating that altered TXNRD1 activity has direct pathological relevance in the nervous system. Hao et al.^19^ further showed that TXNRD1 can drive innate immune signaling under stress conditions. In the present study, this redox-related framework was supported at multiple levels. Single-cell and virtual perturbation analyses converged on redox control and ERK/MAPK-associated immediate-early response programs, and human HSCR tissues showed TXNRD1 upregulation together with selective ERK/MAPK activation. In EGCs, TXNRD1 overexpression increased ROS accumulation and promoted an SPP1-high/CXCL9-low state, whereas NAC blunted ROS elevation, reduced ERK activation, and partially reversed this polarization shift. By contrast, ERK inhibition did not normalize ROS levels. These findings suggest that TXNRD1-associated redox dysregulation is not merely an accompanying event, but may act upstream of ERK/MAPK signaling to promote enteric glial state remodeling in HSCR.

SPP1 is a multifunctional adhesion protein that plays a critical role in disease progression across macrophages, microglia, and tumor ecosystems.^8^^,^^20^^,^^21^ The CXCL9:SPP1 polarization axis is increasingly recognized as a more accurate functional classifier than classical M1/M2 signatures.^22^^,^^23^ Emerging evidence suggests that SPP1^+^ glial populations modulate neuronal survival and plasticity in diverse pathologies.^24^ However, whether a similar CS-like polarization program occurs in EGCs during HSCR and how it contributes to disease progression had remained unclear. In this study, single-cell analysis identified a significantly expanded TXNRD1-enriched, SPP1-enriched EGC subcluster in HSCR, and network-based vOE of TXNRD1 preferentially perturbed SPP1 together with stress-response genes, supporting a pathological SPP1-centered EGC program. Recent work has shown that SPP1-associated glial states are linked to synaptic engulfment and disease-associated plasticity in the nervous system.^25^ Consistent with this view, Al-Dalahmah et al.^26^ demonstrated that SPP1 drives neuroinflammation and neuronal cell loss in frontotemporal dementia patient neurons, highlighting the pathogenic potential of SPP1-associated glial programs in the nervous system. Against this background, our data further showed that TXNRD1 was markedly upregulated in HSCR and closely associated with the SPP1-high glial state. Consistently, TXNRD1 overexpression in EGCs increased SPP1 expression and reduced CXCL9 expression, accompanied by impaired glial migration and reduced neurotrophic support. These findings suggest that TXNRD1 may contribute to HSCR progression by promoting a shift of EGCs toward an SPP1-high/CXCL9-low enteric glial state, although the underlying molecular mechanisms still require further investigation.

A recent study by Gu et al.^27^ demonstrated that SPP1^+^ microglia induce neuronal apoptosis through the Lcn2-SLC22A17 pathway. However, EGCs are traditionally classified into four subtypes based on anatomical location and morphology, making it difficult to precisely distinguish the functional contributions of each subtype to neuronal regulation.^28^ Pang et al.^29^ and Lu et al.^30^ defined EGC activation according to GFAP and S100β expression, showing that ENS injury and altered synaptic plasticity are closely linked to EGC activation. Using single-cell sequencing, Windster et al.^31^ further revealed the presence of canonical and Schwann cell-like EGCs in HSCR patients, with the latter significantly reducing neuronal density. Together, these studies suggest that abnormal EGC activation or polarization exerts substantial deleterious effects on neuronal networks.

To determine whether EGC functional states could be classified through a CS-like polarization framework, we co-cultured primary DRGs with TXNRD1-overexpressing EGCs. In the co-culture system, TXNRD1-overexpressing EGCs increased neuronal apoptosis, impaired neuronal migration, reduced p-RET/RET and GFRα1 expression, downregulated PSD-95 and SYN, and disrupted intracellular Ca^2+^ homeostasis. NGF and BDNF levels were also decreased. Notably, both ERK inhibition and exogenous GDNF partially reversed these abnormalities, supporting the involvement of the GDNF/GFRα1/RET axis in the neuronal defects associated with TXNRD1-overexpressing EGCs.

Building on the single-cell findings described above, GSEA of differentially expressed proteins in our HSCR tissue proteomic dataset further revealed significant enrichment of MAPK pathway. Prior studies have also implicated MAPK signaling in HSCR.^32^^,^^33^ Stavely et al.^34^ showed that ERK inhibition is required for ENCC migration in both mouse and chick models. Consistent with these findings, we observed marked upregulation of ERK/MAPK pathway proteins—including p-Raf, Raf, p-MEK, MEK, p-ERK, and ERK—in HSCR tissues, while p-JNK/JNK and p-p38/p38 remained unchanged. Although this appears to contrast with findings by Wang et al. that EP2-mediated PGE_2_ blockade enhances ENCC migration via p38 activation,^35^ our mechanistic data indicate that TXNRD1 predominantly signals through ERK/MAPK to promote SPP1 expression and suppress CXCL9, and that ERK inhibition reverses these effects. Thus, ERK/MAPK signaling appears to function as a key downstream pathway linking TXNRD1 dysregulation to enteric glial state remodeling in HSCR.

Cell-cell communication plays a critical role in tissue development, homeostasis, and cellular function.^36^ Extensive ligand-receptor interactions occur within the intestinal microenvironment. Zhang et al.^37^ identified glial-neuronal communication mediated by ligand-receptor pairs including CCL2-CCR3 and VCAM1-NCAM1. In HSCR, EGCs have been shown to increase neuronal numbers in aganglionic segments via GDNF-NCAM1 signaling.^38^ Soret et al.^39^ demonstrated that exogenous GDNF prolonged survival, induced enteric neurogenesis, and improved colon structure and function in multiple HSCR mouse models, while also promoting Schwann cell proliferation and new neuron formation in cultured human aganglionic bowel explants. Consistent with these findings, we found that NCAM1 and VCAM1 were significantly downregulated in *Ednrb* cKO mice and were restored by TXNRD1 knockdown. Notably, exogenous GDNF in the DRG-EGC co-culture system partially reversed the neuronal injury caused by TXNRD1-overexpressing EGCs, including increased apoptosis, impaired migration, reduced GFRα1/RET signaling, and synaptic abnormalities. These results collectively suggest that TXNRD1 dysregulation compromises glia-neuron communication and neurotrophic support, thereby exacerbating neuronal dysfunction and HSCR severity. Importantly, these molecular and communication changes were accompanied *in vivo* by attenuation of HSCR-like phenotypes, including improved intestinal morphology, prolonged survival, increased fecal water content, and partial restoration of TUJ1 and p75 expression after TXNRD1 knockdown.

In conclusion, our study identifies TXNRD1 as a central regulator of an SPP1-high/CXCL9-low enteric glial state in HSCR. By integrating single-cell, network-based virtual perturbation, tissue, cellular, and *in vivo* evidence, we show that TXNRD1 is linked to expansion of an SPP1-enriched EGC subcluster, and virtual perturbation analysis further supports TXNRD1 as an upstream regulator of redox- and MAPK-centered glial regulatory remodeling. TXNRD1 drives acquisition of an SPP1-high/CXCL9-low enteric glial state through redox-dependent ERK/MAPK activation. This abnormal glial state is associated with impaired glial migration, reduced neurotrophic support, disrupted GDNF/GFRα1/RET signaling, altered glia-neuron communication, and neuronal dysfunction, and its reversal alleviates HSCR-like phenotypes in *Ednrb* cKO mice. Collectively, these findings establish a mechanistic link between redox dysregulation, enteric glial plasticity, and ENS dysfunction, and nominate TXNRD1 as a potential therapeutic target for HSCR.

### Limitations of the study

This study has several limitations. First, proteomic screening was performed using only two paired HSCR samples, and the validation cohorts of human tissues and *Ednrb* cKO mice were relatively small. Second, the origin and temporal dynamics of the SPP1-high/CXCL9-low EGC state were not resolved, and lineage-tracing and spatial approaches will be required to further characterize this population. Third, primary DRG neurons were used as a surrogate neuronal model, and intraperitoneal AAV-sh-TXNRD1 administration was not restricted to EGCs, limiting a cell-type-specific interpretation of the *in vivo* findings. Fourth, the downstream molecular mechanisms linking the remodeled EGC state to neuronal dysfunction require further investigation. Finally, although both male and female participants and experimental animals were included, the available sample sizes were insufficient for sex-stratified analyses. Future studies with larger cohorts are required to determine whether sex-dependent effects influence TXNRD1-associated enteric glial remodeling in HSCR.

## Resource availability

### Lead contact

Requests for further information, resources, and reagents should be directed to and will be fulfilled or facilitated by the lead contact, Zebing Zheng (zebing1988@sina.com).

### Materials availability

This study did not generate new unique materials or reagents.

### Data and code availability

• The publicly available scRNA-seq dataset analyzed in this study is available in the Gene Expression Omnibus under accession GSE293138. The proteomic data generated in this study have been deposited in iProX under accession PXD072379. The accession numbers are also listed in the key resources table.
• This paper does not report original code.
• Any additional information required to reanalyze the data reported in this paper is available from the lead contact upon request.

## Acknowledgments

This study was supported by the Guizhou Provincial Basic Research Program (Natural Science) (nos. MS-2025–406 and ZK-2024-General-298), the Medical Research Union Fund for High-Quality Health Development of Guizhou Province (no. 2024GZYXKYJJXM0108), and the Doctoral Start-up Foundation of Zunyi Medical University, Guizhou Province (no. 202311).

## Author contributions

Y. Li, conceptualization, methodology, investigation, data curation, visualization, and writing – original draft; Y.W., Z.J., and C.T., methodology, formal analysis, investigation, data curation, and writing – original draft; X.X., Y.G., and Q.D., statistical analysis, animal model construction, and validation; L.H., Z.L., and Y. Liao, clinical sample collection, data acquisition, and validation; S.H., B.W., and Y. Liu, writing – review and editing and supervision. Z.Z., conceptualization, supervision, project administration, and funding acquisition.

## Declaration of interests

The authors declare no competing interests.

## STAR★Methods

### Key resources table

**Table:** 

REAGENT or RESOURCE	SOURCE	IDENTIFIER
**Antibodies**		

Rabbit anti-CXCL9	Invitrogen	Cat# 701117; RRID: AB_2532396
Rabbit anti-p-RET	Invitrogen	Cat# PA5-104769; RRID: AB_2816242
Rabbit anti-GFRα1	Invitrogen	Cat# PA5-120909; RRID: AB_2914481
Rabbit anti-SPP1	Proteintech	Cat# 22952-1-AP; RRID: AB_2783651
Rabbit anti-TXNRD1	Abcam	Cat# ab124954; RRID: AB_10975643
Mouse anti-postsynaptic density protein-95 (PSD-95)	Santa Cruz Biotechnology	Cat# sc-71935
Mouse anti-Synaptophysin (SYN)	Santa Cruz Biotechnology	Cat# sc-365488
Rabbit anti-p75	Abcam	Cat# ab52987; RRID: AB_881682
Mouse anti-beta III Tubulin(TUJ1)	Abcam	Cat# ab78078; RRID: AB_2256751
Rabbit anti-VCAM1	Abcam	Cat# ab134047; RRID: AB_2721053
Mouse anti-NCAM1	Abcam	Cat# ab9018; RRID: AB_306945
Brilliant Violet 421™ AffiniPure® Goat Anti-Mouse IgG (H + L)	Jackson ImmunoResearch	Cat# 115-675-205; RRID: AB_2651088
Goat anti-mouse IgG H&L (Alexa Fluor® 488)	Abcam	AB_2651088Cat# ab150117; RRID: AB_2688012
Goat anti-rabbit IgG H&L (Alexa Fluor® 647)	Abcam	Cat# ab150079; RRID: AB_2722623
Goat anti-rabbit IgG H&L (Alexa Fluor® 488)	Abcam	Cat# ab150077; RRID: AB_2630356
Rabbit anti-ERK1/2	HUABIO	Cat# ET1601-29; RRID: AB_3069611
Rabbit anti-*p*-ERK1/2	HUABIO	Cat# ET1610-13; RRID: AB_3069896
Rabbit anti- MEK1/2	HUABIO	Cat# ET1602-3
Rabbit anti- *p*-MEK1/2	HUABIO	Cat# ET1609-50
Rabbit anti-Raf1	HUABIO	Cat# ET1701-21; RRID: AB_3070189
Rabbit anti-*p*-Raf1	HUABIO	Cat# ET1612-87; RRID: AB_3070164
Rabbit anti-JNK1/2/3	HUABIO	Cat# ET1601-28; RRID: AB_3069610
Rabbit anti-*p*-JNK1/2/3	HUABIO	Cat# ET1609-42; RRID: AB_3069852
Rabbit anti-P38	HUABIO	Cat# HA722036; RRID: AB_3096629
Rabbit anti-p-P38	HUABIO	Cat# ER2001-52; RRID: AB_3069529
Rabbit anti-S100β	HUABIO	Cat# ET1610-3; RRID: AB_3069913
Rabbit anti-RET	HUABIO	Cat# ET1611-98; RRID: AB_3070079
Rabbit anti- Beta Tubulin	HUABIO	Cat# EM0103; RRID: AB_2819165
Rabbit anti- GAPDH	HUABIO	Cat# ET1601-4; RRID: AB_3069615

**Biological samples**		

Paired stenotic and proximal dilated colonic tissues from children with Hirschsprung disease	Affiliated Hospital of Zunyi Medical University	KLL-2023–351 (2023 samples); KLLY-2024–081 (2024 samples)
Animal ethics approval	Affiliated Hospital of Zunyi Medical University	zyfy-an-2023–0074; zyfy-an-2024–0655

**Experimental models: Cell lines**		

EGC/PK060399egfr rat enteric glial cell line	ATCC	CRL-2690

**Experimental models: Organisms/strains**		

*Ednrb* floxed mouse	Shanghai Model Organisms Center	C57BL/6Smoc-*Ednrb*^*tm1(flox)Smoc*^
*Wnt1-*Cre mouse	The Jackson Laboratory	129S4.Cg-*E2f1*^*Tg(Wnt1-cre)2Sor*^/J(Jax:022137)
Sprague–Dawley rat pups for primary DRG culture	Laboratory Animal Center of Zunyi Medical University	Vendor-derived SD rats, postnatal day 0–2

**Chemicals, peptides, and recombinant proteins**		

SCH772984	ALTPHOSO™ Haotianqi Bio	HTQ-YW-26803
N-acetyl-L-cysteine (NAC)	Sigma-Aldrich	A7250
Recombinant rat GDNF	Beyotime	P6411
DMSO	ALTPHOSO™ Haotianqi Bio	HTQ-YW-61041

**Critical commercial assays**		

CCK-8 assay	Beyotime	C0039
Annexin V-FITC/PI apoptosis assay	Servicebio	G1511
ROS assay kit	MedChemExpress	HY-K0320
Micro TrxR assay kit	Abbkine	KTB1650
Fluo-4 calcium assay kit	Beyotime	S1061
Rat GDNF ELISA kit	Abcam	ab213901
Rat TNF-α ELISA kit	Beyotime	PT516
Rat NGF ELISA kit	ALTPHOSO™ Haotianqi Bio	HTQ-ES-02145
Rat BDNF ELISA kit	ALTPHOSO™ Haotianqi Bio	HTQ-ES-02165

**Recombinant DNA**		

LV-OE-TXNRD1	GenePharma	This paper
LV-OE-NC	GenePharma	This paper
pAAV-U6-shRNA(TXNRD1)-CMV-ZsGreen1-WPRE	GenePharma	This paper
pAAV-U6-shRNA(NC)-CMV-ZsGreen1-WPRE	GenePharma	This paper

**Deposited data**		

Publicly available HSCR scRNA-seq dataset	GEO	GSE293138
Proteomic dataset generated in this study	iProX	PXD072379

**Software and algorithms**		

Seurat	Hao et al.^40^	v4.3.3
Harmony	Korsunsky et al.^41^	v1.2.0
CellChat	Jin et al.^42^	v1.6.1
scTenifoldNet	Osorio et al.^43^	v1.3
clusterProfiler	Wu et al.^44^	v4.12.0
fgsea	Korotkevich	v1.28.0
ggplot2	Wickham	v3.5.1
Spectronaut	Biognosys	v18
GraphPad Prism	GraphPad Software	v9.0
R	R Core Team	v4.3.2
ImageJ	National Institutes of Health	v1.54f

**Oligonucleotides**		

Primers used for RT-qPCR and genotyping	This paper	See Table S1

### Experimental model and study participant details

#### Human participants

Paired colonic tissue samples were collected from eight children with Hirschsprung disease who underwent definitive pull-through surgery at the Affiliated Hospital of Zunyi Medical University between June 2023 and December 2024. The cohort included 4 male and 4 female participants, with a median age at surgery of 3 months.

For each participant, the stenotic aganglionic segment and a proximal dilated ganglionic segment were collected intraoperatively. The proximal dilated ganglionic segment was used as a paired within-patient comparator and was not considered healthy control colon. Two paired tissue samples were selected for exploratory proteomic profiling, whereas all eight paired samples were used for tissue-level validation experiments.

The diagnosis of Hirschsprung disease was established on the basis of clinical manifestations, radiological findings, operative findings, and postoperative histopathological confirmation. Inclusion criteria were as follows: (1) histopathological and radiographic confirmation of Hirschsprung disease; (2) fulfillment of the clinical diagnostic criteria for Hirschsprung disease; (3) complete clinical records; and (4) written informed consent from a legal guardian. Exclusion criteria were as follows: (1) renal insufficiency; (2) additional congenital disorders; (3) digestive tract malignancy; (4) coagulation disorders; or (5) concurrent necrotizing enterocolitis.

The study protocols covering the entire sample collection period from June 2023 to December 2024 were approved by the Ethics Committee of the Affiliated Hospital of Zunyi Medical University (KLL-2023–351 for 2023 samples and KLLY-2024–081 for 2024 samples) and were conducted in accordance with the Declaration of Helsinki. Written informed consent was obtained from the legal guardian of each participant before sample collection. Sex was recorded for all participants; however, sex-specific analyses were not performed because of the limited sample size. The potential influence of sex on the interpretation and generalizability of the findings is acknowledged in the limitations of the study.

#### Experimental animals

C57BL/6Smoc-*Ednrb*^*tm1(flox)Smoc*^ purchased from Shanghai Model Organisms Center(China), and the 129S4.Cg-*E2f1*^*Tg(Wnt1-cre)2Sor*^/J(Jax:022137) purchased from The Jackson Laboratory (USA). *Ednrb* cKO mice (*Wnt1*^cre/-^ *Ednrb*^flox/flox^) and littermate controls were generated as described below. For AAV-mediated intervention, postnatal day 5 mice were used. Both male and female mice were used in the *in vivo* experiments. Mice were housed in a specific pathogen-free (SPF) barrier facility under controlled environmental conditions (22 ± 2°C, 50 ± 10% relative humidity, 12-h light/12-h dark cycle) with free access to food and water. All animals were routinely monitored according to institutional animal health surveillance standards and were confirmed to be free of specific pathogens. All experimental procedures were conducted under protocols approved by the Laboratory Animal Welfare and Ethics Committee of the Affiliated Hospital of Zunyi Medical University (approval numbers: zyfy-an-2023–0074 and zyfy-an-2024–0655).

#### Cell line

The rat enteric glial cell line EGC/PK060399egfr was obtained from the American Type Culture Collection (ATCC; CRL-2690). This cell line was originally isolated from the jejunum of an adult male rat. Cell identity was confirmed by immunofluorescence staining for the enteric glial marker S100β (Figure S3J). No additional genetic authentication was performed in our laboratory. The cells were not tested for mycoplasma contamination.

#### Primary dorsal root ganglion cultures

Primary dorsal root ganglion neurons were isolated from neonatal SD rat pups at postnatal day 0–2. Rats were obtained from the Laboratory Animal Center of Zunyi Medical University and maintained under SPF conditions before tissue collection. Both male and female pups were used.

All procedures involving neonatal rats were approved by the Laboratory Animal Welfare and Ethics Committee of the Affiliated Hospital of Zunyi Medical University (approval numbers: zyfy-an-2023–0074 and zyfy-an-2024–0655).

#### Sex as a biological variable

Sex was considered as a biological variable throughout the study. Human samples included both male and female patients, and both male and female mice and neonatal rats were included in experimental models. Because the available sample sizes were not designed to detect sex-dependent differences, sex-stratified statistical analyses were not performed. The potential effects of sex on the interpretation and generalizability of the findings are acknowledged in the Limitations of the Study.

### Method details

#### Acquisition and processing of scRNA-seq data

The publicly available scRNA-seq dataset GSE293138 was obtained from the GEO database and comprised eight colonic tissue samples from four 10–14-day-old mice: proximal and distal colon from two wild-type mice and two *Ednrb*-null HSCR-model mice. Data were processed using Seurat v4.3.3. Cells expressing 200–6,000 genes with <20% mitochondrial unique molecular identifiers were retained, and mitochondrial genes were removed before downstream analysis. The top 2,000 highly variable genes were identified using the “vst” method, followed by data scaling and principal component analysis. Batch effects were corrected using Harmony with default parameters. Cells were clustered using FindClusters at a resolution of 0.5 and visualized using UMAP. Cell populations were annotated according to cluster-specific markers and established canonical markers.

#### Cell communication analysis

Intercellular communication was inferred using CellChat (v1.6.1), which predicts ligand–receptor interactions based on curated databases. Communication probabilities were calculated between enteric glial cell subclusters and enteric neurons using default parameters unless otherwise specified. Signaling pathway strength and interaction networks were visualized based on aggregated communication probability scores. All inferred interactions represent computational predictions and not experimentally validated ligand–receptor binding.

#### Identification of differentially expressed genes

Differentially expressed genes between selected cell clusters were identified using the FindMarkers function in Seurat with the Wilcoxon rank-sum test. Genes with |logFC| > 0.25, expression in >25% of cells in the target cluster (min.pct >0.25), and adjusted *p* < 0.05 were considered significant. *p* values were adjusted using the Benjamini–Hochberg method.

#### Gene functional enrichment analysis

Gene Ontology (GO) biological process and Kyoto Encyclopedia of Genes and Genomes (KEGG) pathway enrichment analyses were performed using the R package clusterProfiler. Gene sets with adjusted *p* < 0.05 and false discovery rate (FDR) < 0.25 were considered significantly enriched. Enrichment results were visualized using ggplot2 (v3.5.1) in R.

#### Construction of the wild-type single-cell gene regulatory network

To evaluate regulatory network changes associated with TXNRD1, gene regulatory networks were constructed using the scTenifoldNet framework.

Briefly, a WT single-cell gene expression matrix was used to construct multiple subsampled networks using the makeNetworks function. Ten independent subnetworks (nNet = 10) were generated by randomly sampling 500 cells per iteration (nCells = 500), retaining the top three principal components (nComp = 3). Parallel computation was enabled using all available CPU cores. The resulting network tensor was integrated and denoised using CANDECOMP/PARAFAC tensor decomposition (K = 3) to generate the final WT gene regulatory network.

#### Virtual perturbation of TXNRD1

To evaluate the regulatory influence of Txnrd1 within the inferred WT network, virtual knockout (vKO) and virtual overexpression (vOE) analyses were performed at the network level. For vKO, all outgoing regulatory effects of the Txnrd1 node were removed by setting the corresponding row of the WT network matrix to zero, thereby simulating the loss of Txnrd1-mediated regulatory activity.

For vOE, all outgoing regulatory weights of the Txnrd1 node were multiplied by a fixed scaling factor, thereby simulating enhanced Txnrd1-mediated regulatory activity. The resulting vKO and vOE networks were separately compared with the original WT network to characterize perturbation-associated changes in regulatory network structure.

These procedures were performed exclusively in silico on the inferred gene regulatory network. Therefore, vKO and vOE represent network-level virtual perturbations and should not be interpreted as experimental gene knockout or overexpression.

#### Manifold alignment and identification of differentially regulated genes

WT and perturbed networks were compared using manifold alignment implemented in scTenifoldNet. Differential regulation was quantified by computing Euclidean distances between each gene in the WT-aligned manifold and its corresponding gene in the perturbed manifold. Distance values were transformed using Box–Cox transformation when appropriate and standardized into Z-scores. A chi-square distribution with 1° of freedom was used to estimate statistical significance. *p* values were adjusted using the Benjamini–Hochberg method. Genes with significant adjusted *p* values and high Z-scores were defined as differentially regulated under TXNRD1 perturbation.

#### Protein extraction and trypsin digestion

Tissue samples were pulverized in liquid nitrogen and precipitated using pre-chilled 10% trichloroacetic acid in acetone at −20°C for 4 h. Protein pellets were washed three times with cold acetone and resuspended in lysis buffer (8 M urea, 1% protease inhibitor cocktail). Samples were sonicated on ice and centrifuged at 12,000×g for 10 min at 4°C. Supernatants were collected and protein concentration was determined using a BCA assay. Proteins were reduced with 5 mM dithiothreitol (30 min, 56°C) and alkylated with 11 mM iodoacetamide (15 min, dark, room temperature). Trypsin digestion was performed at a 1:50 enzyme-to-protein ratio overnight at 37°C. Peptides were desalted using Strata-X solid-phase extraction columns.

#### LC-MS/MS Analysis

Peptides were dissolved in solvent A (0.1% formic acid, 2% acetonitrile) and separated using an Easy-nLC1000 UHPLC system at 500 nL/min with a linear gradient of solvent B (0.1% formic acid in acetonitrile). Mass spectrometry was performed on a timsTOF Pro instrument operating in DIA-PASEF mode. MS1 scans were acquired over 300–1500 m/z with 20 PASEF MS/MS scans per cycle (MS2 range: 400–850 m/z). Spectral libraries were generated using Spectronaut v18 against the Homo sapiens UniProt database (2023 release). Carbamidomethylation of cysteine was set as a fixed modification, while methionine oxidation and N-terminal acetylation were set as variable modifications. A maximum of two missed cleavages was allowed. Protein, peptide, and PSM-level false discovery rate (FDR) was set to 1%.

#### Gene set enrichment analysis (GSEA)

GSEA was performed using the fgsea package in R based on proteomic differential expression results. Proteins were ranked according to differential abundance between stenotic and paired proximal dilated colonic tissues. Enrichment analysis was performed against KEGG pathway gene sets. Visualization was performed using ggplot2 (v3.5.1). Pathways with nominal *p* < 0.05 and FDR <0.25 were considered significantly enriched.

#### Immunofluorescence staining

For tissue immunofluorescence staining, paraffin sections were deparaffinized in xylene and rehydrated through graded ethanol. Antigen retrieval was performed in sodium citrate buffer (pH 6.0; Meilunbio) using microwave heating for 20 min. Sections were blocked with 3% bovine serum albumin at room temperature for 1 h and incubated with primary antibodies at 4°C overnight. After washing with phosphate-buffered saline, sections were incubated with species-appropriate fluorescent secondary antibodies for 1 h at room temperature in the dark. Nuclei were counterstained with DAPI, and sections were mounted with antifade mounting medium.

For cell immunofluorescence staining, cells were seeded on sterile coverslips in 24-well plates. Cells were fixed with 75% ethanol for 10 min at room temperature, permeabilized with 0.1% Triton X-100 for 10 min, and blocked with 3% bovine serum albumin for 1 h. Cells were then incubated with primary antibodies at 4°C overnight, followed by fluorescent secondary antibodies. Nuclei were stained with DAPI for 5 min.

Images were acquired using an Olympus fluorescence microscope. For each experiment, images from comparison groups were acquired using identical exposure settings. Quantification was performed in 3 randomly selected non-overlapping fields per tissue sample, mouse, or independent cell culture using ImageJ. Investigators performing image quantification were blinded to group allocation.

#### Western blot

Total proteins were extracted from cells and tissues using RIPA lysis buffer. Protein concentrations were measured using a BCA assay. Equal amounts of protein (10 μg per lane) were denatured by heating at 100°C for 10 min, separated by SDS-PAGE, and transferred to polyvinylidene difluoride membranes.

Membranes were blocked with Protein-Free Rapid Blocking Buffer for 15 min at 25°C and incubated with primary antibodies overnight at 4°C. After washing with TBST, membranes were incubated with horseradish peroxidase-conjugated secondary antibodies for 1 h at room temperature. Protein bands were visualized using enhanced chemiluminescence reagent and imaged using a Tanon-4600 imaging system.

Band intensities were quantified using ImageJ. Target-protein signals were normalized to the corresponding loading controls or total protein signals. Target proteins and loading controls were derived from the same membrane unless otherwise indicated in the uncropped raw data file. Molecular weight markers, full membrane images, the exact bands used for quantification, and the relationship between each target protein and its loading control are provided in the uncropped blot source data.

#### RT-qPCR

Total RNA was extracted from tissues or cells using TRIzol reagent (Invitrogen, USA). Reverse transcription was performed using PrimeScript RT Master Mix (RR036Q, Takara, Japan) to synthesize cDNA. Next, the cDNA fragments were amplified and analyzed on an ABI 7500 PCR system (ThermoFisher Scientific, USA). Relative expression of the target genes was calculated using the 2^−ΔΔCt^ method, with GAPDH used as the endogenous reference gene for normalization. The primer sequences used are detailed in Table S1.

#### Lentiviral transduction and pharmacological treatments

TXNRD1-overexpressing lentivirus (LV-OE-TXNRD1) and negative-control lentivirus (LV-OE-NC) were obtained from GenePharma. EGCs were transduced according to the manufacturer’s instructions. At 48 h after transduction, cells were subjected to downstream analyses or pharmacological treatments.

For ERK pathway inhibition, SCH772984 was used at a final concentration of 10 nM for 24 h. For antioxidant treatment, NAC was used at a final concentration of 1 mM for 24 h. For rescue experiments, recombinant GDNF was added at a final concentration of 50 ng/mL for 24 h. Vehicle controls received equivalent concentrations of DMSO.

#### Cell culture and treatment

The rat enteric glial cell line EGC/PK060399egfr was cultured in DMEM/F-12 medium supplemented with 10% fetal bovine serum, 100 U/mL penicillin, and 100 μg/mL streptomycin at 37°C in a humidified incubator containing 5% CO_2_.

#### DRG-EGC transwell co-culture

For routine non-contact co-culture experiments, EGCs were seeded in the upper compartment of Transwell inserts with 8-μm pores (Corning), whereas primary DRG cells were seeded in the lower compartment. For migration assays, the positions of the two cell types were adjusted according to the cell type being evaluated, as described below. EGCs were transduced with LV-OE-TXNRD1 or LV-OE-NC before co-culture and were treated with SCH772984, NAC, recombinant GDNF, or vehicle as indicated. DRG cells and EGCs were co-cultured for 24 h before collection for subsequent analyses.

#### Primary DRG isolation and culture

Primary DRG neurons were isolated from neonatal Sprague–Dawley rat pups at postnatal day 0–2. For each independent biological replicate, DRGs were isolated from multiple pups derived from the same litter and pooled for culture. Each independent experiment used DRGs obtained from separate litters to ensure biological independence. DRGs were maintained in specialized rat DRG medium (IMP-R153-1, IMMOCELL) at 37°C in a humidified incubator containing 5% CO_2_.

Neonatal rats were disinfected with 75% ethanol and euthanized by cervical dislocation. The spinal cord was exposed, and dorsal root ganglia were isolated into pre-cooled phosphate-buffered saline. Surrounding connective tissue was removed, and DRGs were digested with 0.25% trypsin at 37°C for 30 min.

Digestion was terminated using DMEM/F-12 medium supplemented with 10% fetal bovine serum, 100 U/mL penicillin, and 100 μg/mL streptomycin. Tissue was gently triturated 10–15 times using a 1-mL pipette. The cell suspension was filtered through a 70-μm nylon mesh, centrifuged at 1,000 rpm for 5 min, and resuspended in specialized rat DRG medium. Cells were adjusted to a density of 5 × 10^5^ cells/mL and seeded on poly-L-lysine-coated six-well plates. The culture medium was replaced after 24 h and every 2–3 days thereafter.

#### Cell proliferation, migration, apoptosis, ROS, TrxR activity, and calcium assays

Cell viability was assessed using a CCK-8 assay according to the manufacturer’s instructions. EGCs were seeded in 96-well plates at 8 × 10^3^ cells per well and treated with the indicated concentrations of SCH772984 for 24 h. CCK-8 reagent was then added at 10 μL per 100 μL of culture medium, and the cells were incubated for 1 h at 37°C before absorbance was measured at 450 nm.

Cell migration was assessed using Transwell inserts with 8-μm pores (Corning). For each assay, the cell type being evaluated was seeded in the upper compartment at 5 × 10^4^ cells per insert, whereas the interacting cell type was seeded in the lower compartment. Accordingly, EGCs were placed in the upper compartment for EGC migration assays, whereas DRG cells were placed in the upper compartment for neuronal migration assays. After 24 h, non-migrated cells on the upper surface of the membrane were removed, and cells that had migrated to the lower surface were fixed, stained with crystal violet, imaged, and quantified.

Apoptosis was quantified using the Annexin V-FITC/PI Cell Apoptosis Detection Kit and analyzed using a BD FACSCelesta FCM. Intracellular ROS levels were measured using a ROS assay kit and analyzed using the same FCM. Intracellular calcium levels were measured using the Fluo-4 Calcium Assay Kit.

TrxR activity was measured using a Micro TrxR Assay Kit according to the manufacturer’s instructions. Absorbance was measured at 412 nm, and TrxR activity was normalized to total protein concentration.

#### Generation and genotyping of *Ednrb* cKO mice

To generate *Ednrb* cKO mice,the two mice were mated with each other to obtain F1 mice of *Wnt1*^cre/-^ *Ednrb*^flox/-^ and *Ednrb*^flox/flox^, and then the F1 mice were crossed to obtain *Ednrb* cKO mice(*Wnt1*^cre/-^ *Ednrb*^flox/flox^), and littermate *Ednrb* ^±^ mice served as control. All mice were genotyped using the Mouse Tail Genotype Rapid Identification Kit(D7283S, Beyotime, China) and agarose gel electrophoresis. Primer information is shown in Table S1.

#### *In vivo* AAV-mediated TXNRD1 knockdown

For *in vivo* TXNRD1 knockdown, postnatal day 5 mice received intraperitoneal injections of AAV vectors expressing TXNRD1 shRNA [pAAV-U6-shRNA(TXNRD1)-CMV-ZsGreen1-WPRE] or negative-control shRNA [pAAV-U6-shRNA(NC)-CMV-ZsGreen1-WPRE]. Each mouse received 2 × 10^11^ viral particles in 200 μL phosphate-buffered saline.

Mice were monitored daily for body weight, abdominal distension, fecal retention, activity, and general condition. Humane endpoints were predefined as abdominal distension, fecal retention, lethargy, and marked weight loss. Mice meeting humane endpoint criteria were euthanized by intraperitoneal administration of an overdose of 1% pentobarbital sodium (0.3 mL/20 g), followed by collection of stenotic intestinal segments for downstream analyses.

#### Fecal water content

Fresh fecal pellets were collected from individually housed mice over a fixed 4-h period. The wet weight was measured immediately after collection. Samples were dried at 60 °C until a constant weight was reached, and fecal water content was calculated using the following equation:

$$\text{Fecal}\;\text{water}\;\text{content}\;\left(\%\right)=\frac{\text{wet}\;\text{weight}-\text{dry}\;\text{weight}}{\text{wet}\;\text{weight}}\times 100$$

#### ELISA

GDNF, TNF-α, NGF, and BDNF concentrations in culture supernatants were measured using commercial rat ELISA kits according to the manufacturers’ instructions. Absorbance was measured at 450 nm using a Bio-Rad microplate reader. Concentrations were calculated using standard curves and normalized to volume.

### Quantification and statistical analysis

Statistical analyses were performed using GraphPad Prism 9.0 and R version 4.3.2. Unless otherwise indicated, quantitative data are presented as mean ± SD. The exact definition of n, number of independent biological replicates, statistical test, center and dispersion measures, and number of independent experimental repeats are provided in the corresponding figure legends. Individual data points are shown where applicable.

For paired comparisons between stenotic and proximal dilated colonic tissues obtained from the same patient, paired two-tailed Student’s t tests were used. For comparisons between two independent groups, unpaired two-tailed Student’s t tests were used. For comparisons among three or more groups, one-way ANOVA followed by Tukey’s multiple-comparison test was used. Survival distributions were compared using the log rank test.

For scRNA-seq analyses, differential gene expression was assessed using the Wilcoxon rank-sum test implemented in Seurat. *p* values from differential-expression and enrichment analyses were adjusted using the Benjamini–Hochberg method where indicated. Proximal and distal colonic samples obtained from the same mouse in GSE293138 were not treated as independent animal replicates. EGC subcluster proportions were summarized descriptively by genotype without inferential statistical testing. Statistical significance is indicated as ∗*p* < 0.05, ∗∗*p* < 0.01, and ∗∗∗*p* < 0.001; ns, not significant.
