# Supplementary material for: TXNRD1-driven enteric glial remodeling promotes neuronal dysfunction in Hirschsprung disease
Source: iScience. 2026 Oct 1;29(10):117636. doi: 10.1016/j.isci.2026.117636 (PMC13657354; doi:10.1016/j.isci.2026.117636)
Supplement: Supplementary file 1 [file mmc1.pdf]

iScience, Volume *29*

Supplemental information

TXNRD1-driven enteric glial remodeling promotes neuronal dysfunction in Hirschsprung disease

Yanyi Li, Yuanli Wang, Zhu Jin, Chengyan Tang, Xinrong Xia, Yuan Gong, Qing Du, Lu Huang, Zeping Li, Yu Liao, Shengyin He, Bo Wang, Yuanmei Liu, and Zebing Zheng

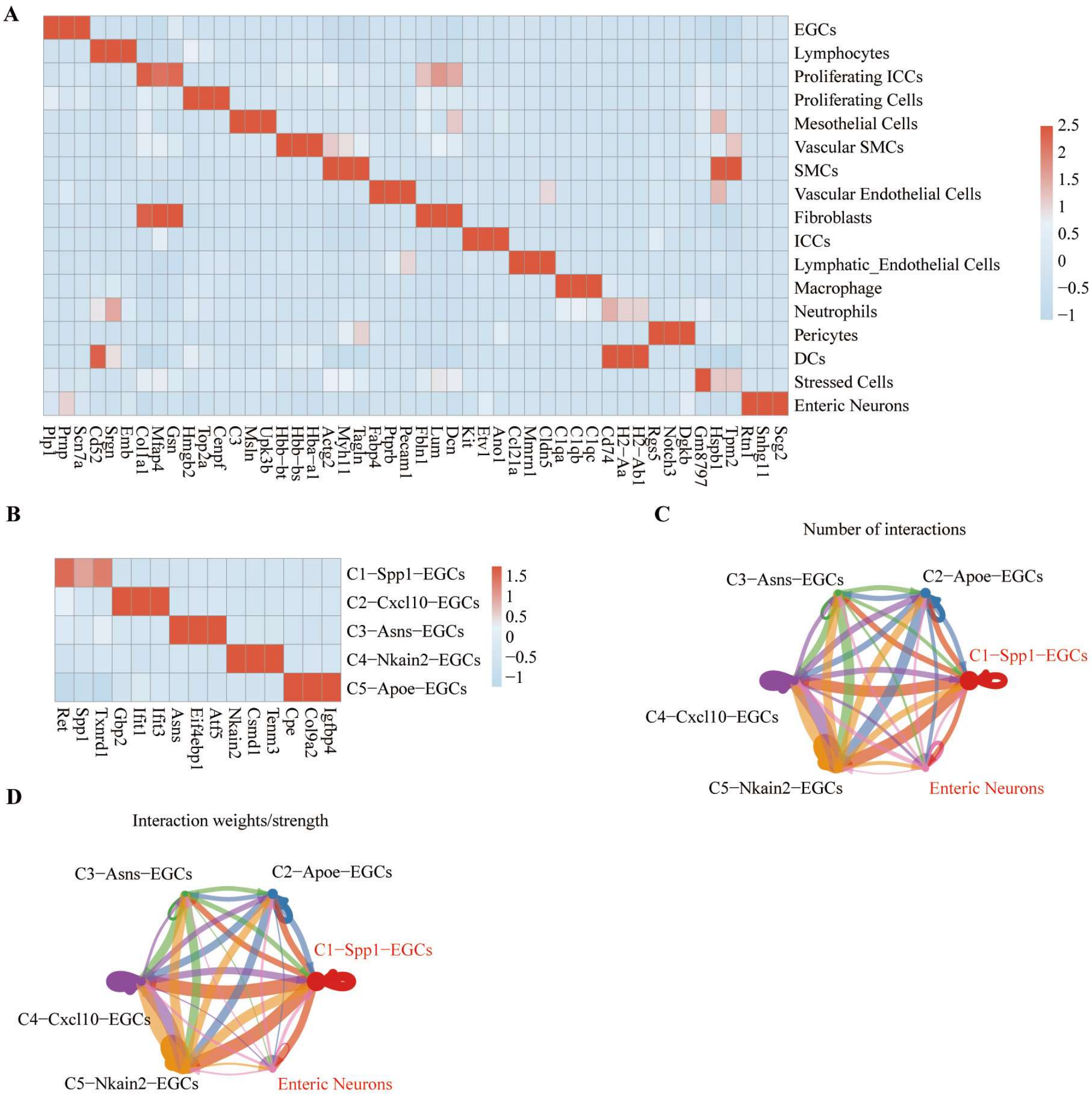

Figure S1 Marker-gene expression and CellChat analysis define EGC subclusters and their predicted communication with enteric neurons

The scRNA-seq dataset GSE293138 included eight colonic tissue samples derived from four mice: proximal and distal colon from two WT mice and two Ednrb-null HSCR-model mice.

A: Heatmap showing representative marker-gene expression across the major intestinal cell populations;

B: Heatmap showing representative marker-gene expression across the five EGC subclusters;

C: CellChat circle plot showing the predicted number of interactions among EGC

subclusters and enteric neurons;

D: CellChat circle plot showing the predicted interaction weights or strengths among EGC subclusters and enteric neurons

The dataset comprised four tissue samples per genotype derived from two mice per genotype, with proximal and distal colon collected from each mouse. Heatmap values represent scaled cell-level gene expression, and CellChat values represent inferred ligand–receptor communication events.

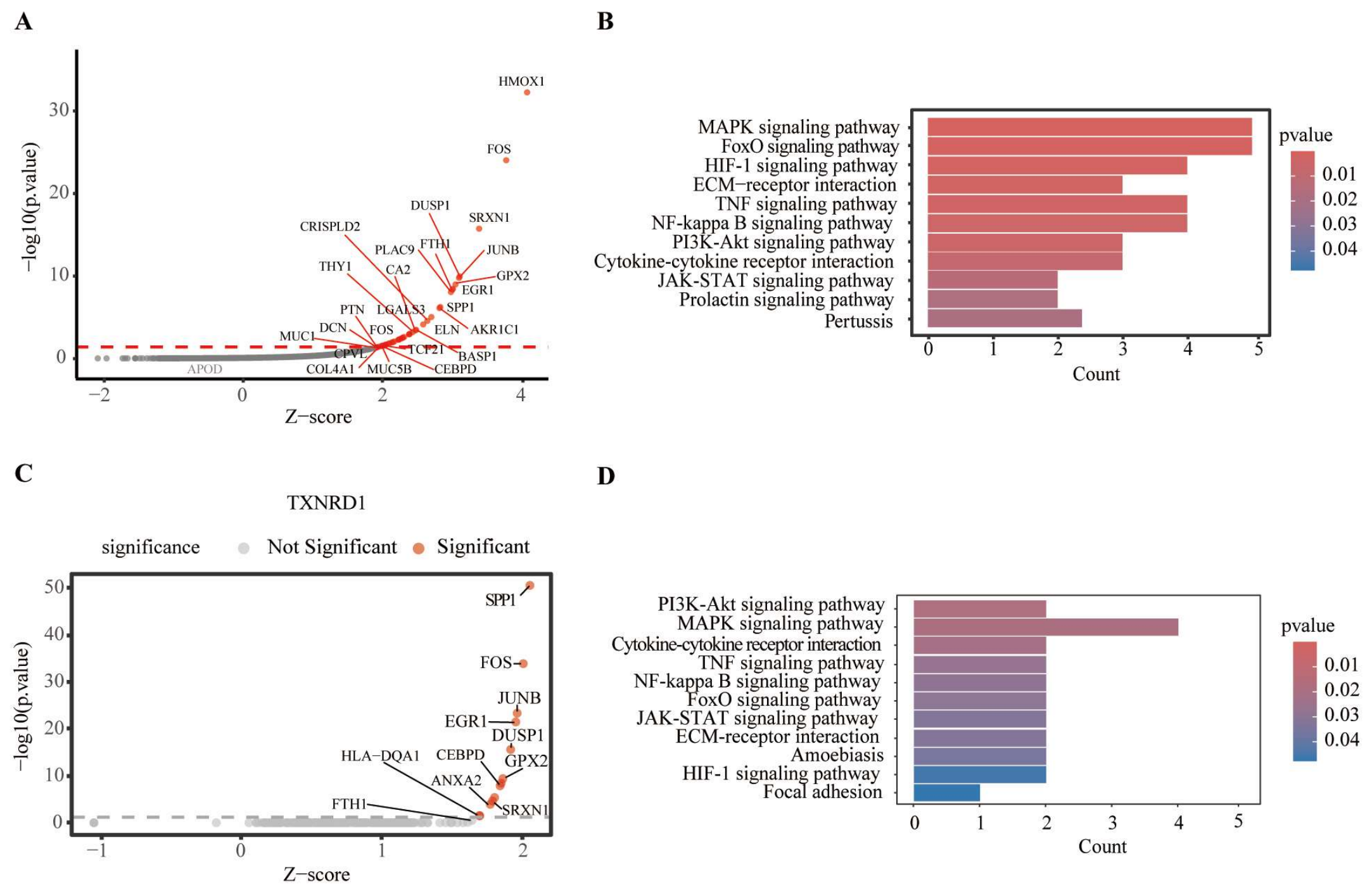

Figure S2 Virtual TXNRD1 perturbation identifies redox- and MAPK-centered regulatory programs in EGCs

Virtual perturbation was performed within the WT EGC regulatory network inferred using scTenifoldNet. For vKO, all outgoing regulatory effects of the TXNRD1 node were set to zero. For vOE, all outgoing regulatory weights of the TXNRD1 node were multiplied by a fixed scaling factor.

A: Differential-regulation plot after TXNRD1 vKO. Each point represents one gene in the aligned regulatory manifold; significantly perturbed genes are highlighted;

B: KEGG enrichment analysis of genes significantly perturbed by TXNRD1 vKO;

C: Differential-regulation plot after TXNRD1 vOE, highlighting *Spp1* and stress-response-associated genes;

D: KEGG enrichment analysis of genes significantly perturbed by TXNRD1 vOE.

Differential regulation was quantified using Euclidean distances between

corresponding genes in the aligned WT and perturbed manifolds. Distances were Box-Cox transformed when applicable and standardized as Z-scores. P values were estimated using a chi-square distribution with one degree of freedom and adjusted using the Benjamini-Hochberg method. Adjusted $P < 0.05$ was considered statistically significant. Virtual perturbations represent computational network simulations rather than experimental genetic manipulation.

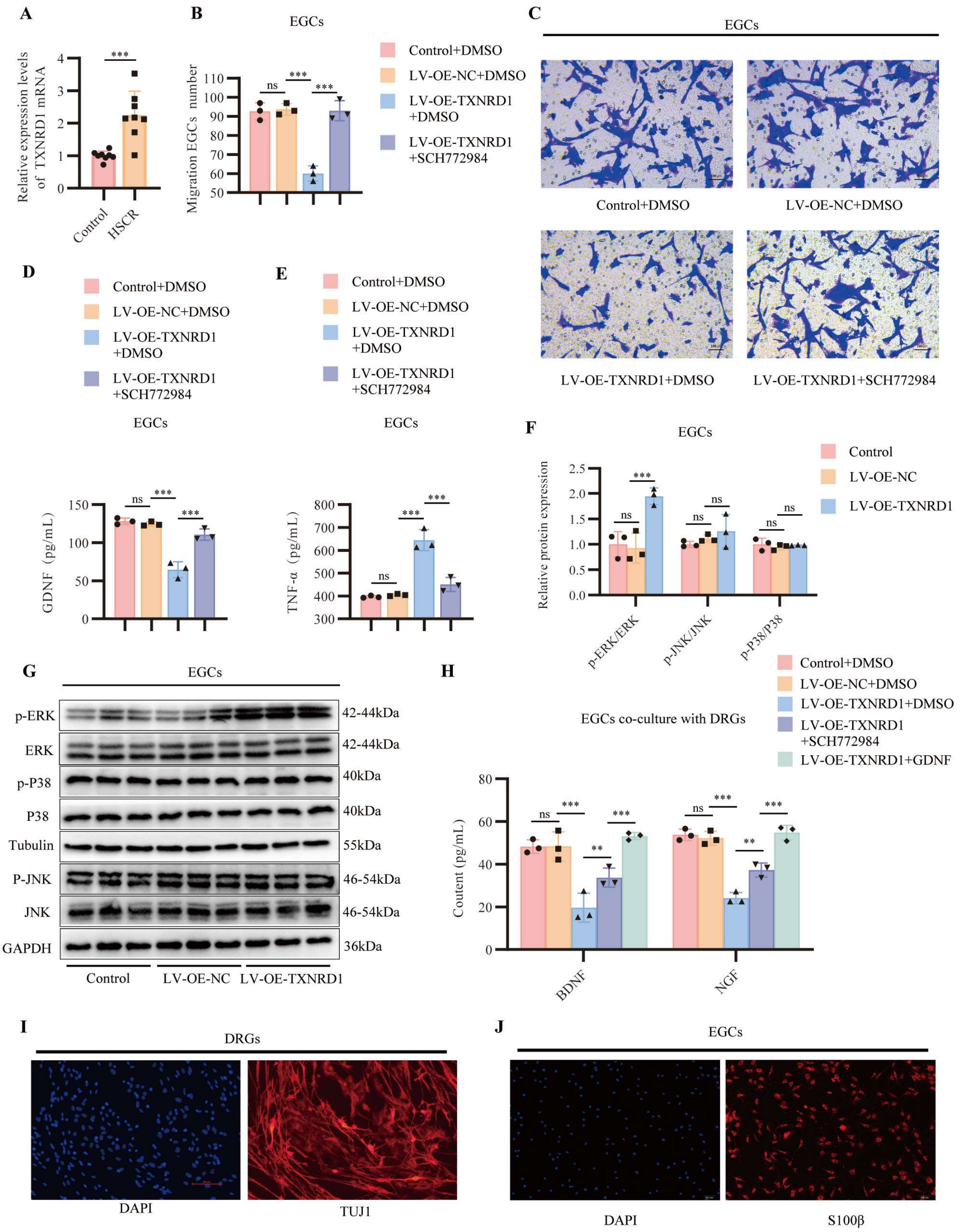

Figure S3 TXNRD1 overexpression impairs EGC migration, alters neurotrophic-factor secretion, and selectively activates ERK signaling.

A: RT-qPCR analysis of TXNRD1 mRNA in paired proximal dilated and stenotic colonic tissues from children with HSCR;

For (B)-(E), cultured rat EGCs were assigned to Control+DMSO, LV-OE-NC+DMSO,

LV-OE-TXNRD1+DMSO, and LV-OE-TXNRD1+SCH772984 groups.

B-C: Quantification (B) and representative crystal-violet images (C) of migrated EGCs;

D-E: ELISA measurements of GDNF (D) and TNF-α (E) in EGC culture supernatants;

F-G: Densitometric quantification (F) and representative WB (G) of p-ERK/ERK, p-JNK/JNK, and p-p38/p38 in Control, LV-OE-NC, and LV-OE-TXNRD1 EGCs;

H: ELISA measurements of BDNF and NGF in supernatants from the five DRG-EGC co-culture groups described in Figure 5;

I: Representative IF images confirming TUJ1 expression in primary DRG cultures;

J: Representative IF images confirming S100β expression in EGC cultures.

For (A), n=8 paired patient samples; each dot represents one patient, and a paired two-tailed Student's t test was used. For cell-based assays, n=3 independent biological experiments; each dot represents one independent experiment. Data are presented as mean ± SD with individual data points. One-way ANOVA followed by Tukey's multiple-comparison test was used for (B), (D)–(F), and (H). *P < 0.05, **P < 0.01, ***P < 0.001; ns, not significant. Crystal-violet images, blots, and micrographs are representative of three independent experiments. Scale bars, 100 μm in (C), (I), and (J).

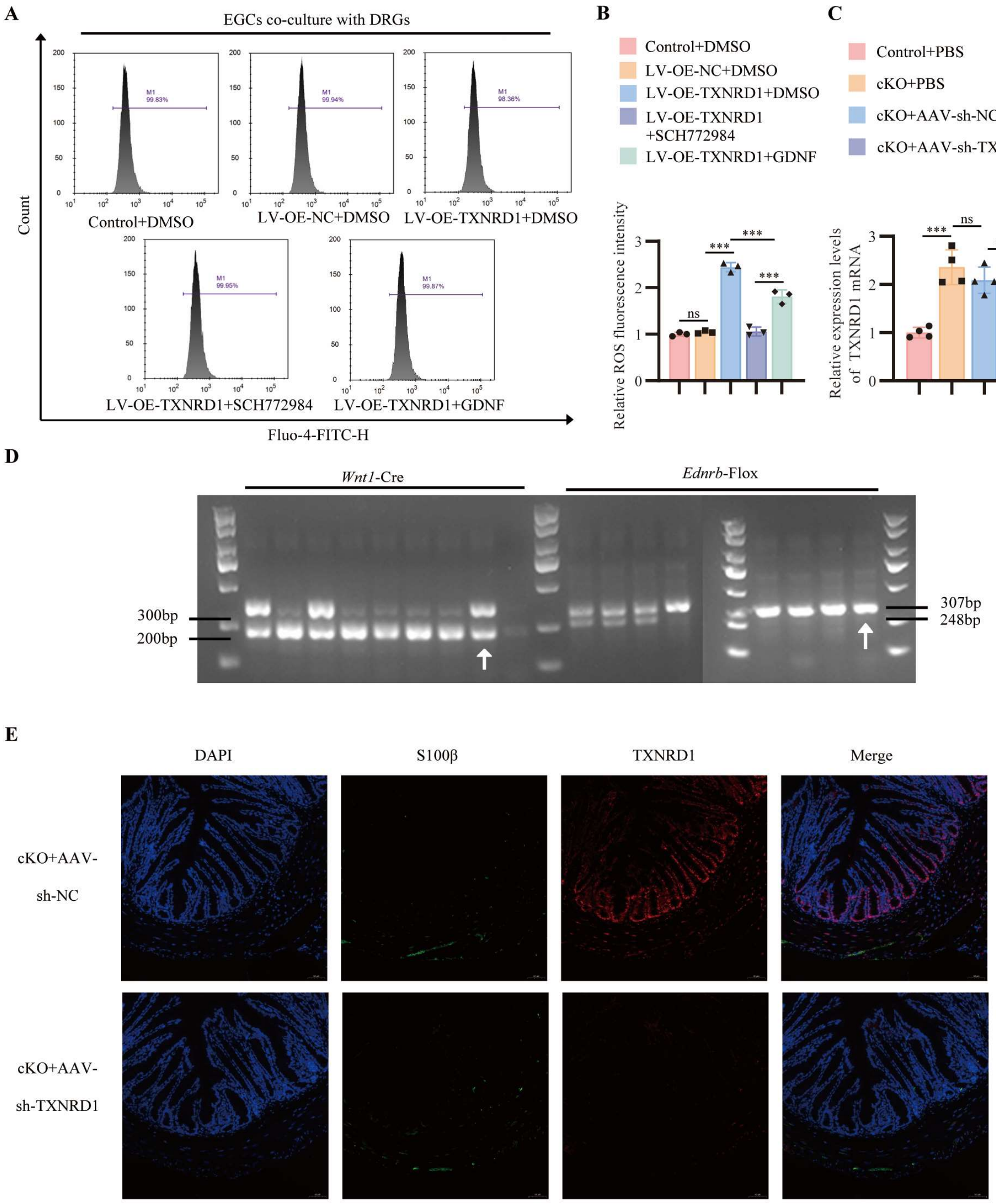

Figure S4 GDNF and ERK inhibition partially restore DRG calcium homeostasis, and AAV-sh-TXNRD1 reduces TXNRD1 expression in *Ednrb* cKO mice

A-B: Representative Fluo-4 FCM histograms of intracellular Ca^{2+} in DRGs from the five DRG–EGC co-culture groups described in Figure 5 (A) and quantification of relative fluorescence intensity (B);

C: RT-qPCR analysis of *Txnrd1* mRNA in intestinal tissues from Control + PBS, cKO + PBS, cKO + AAV-sh-NC, and cKO + AAV-sh-TXNRD1 mice;

D: Representative agarose gel electrophoresis of PCR products used to genotype *Wnt1*-Cre and *Ednrb*-flox alleles. Molecular sizes are indicated, and white arrows mark the cKO genotype;

E: Representative IF images of TXNRD1 and S100β in intestinal tissues from cKO+AAV-sh-NC and cKO+AAV-sh-TXNRD1 mice.

For (A) and (B), n=3 independent DRG–EGC co-culture experiments; each dot represents one independent experiment. For (C), n=3 mice per group; each dot represents one mouse. For (E), images are representative of n=4 mice per group. Quantitative data are presented as mean ± SD with individual data points. One-way ANOVA followed by Tukey's multiple-comparison test was used for (B) and (C). **P < 0.01, ***P < 0.001; ns, not significant. Flow-cytometry plots are representative of three independent experiments. Scale bars, 50 μm in (E).

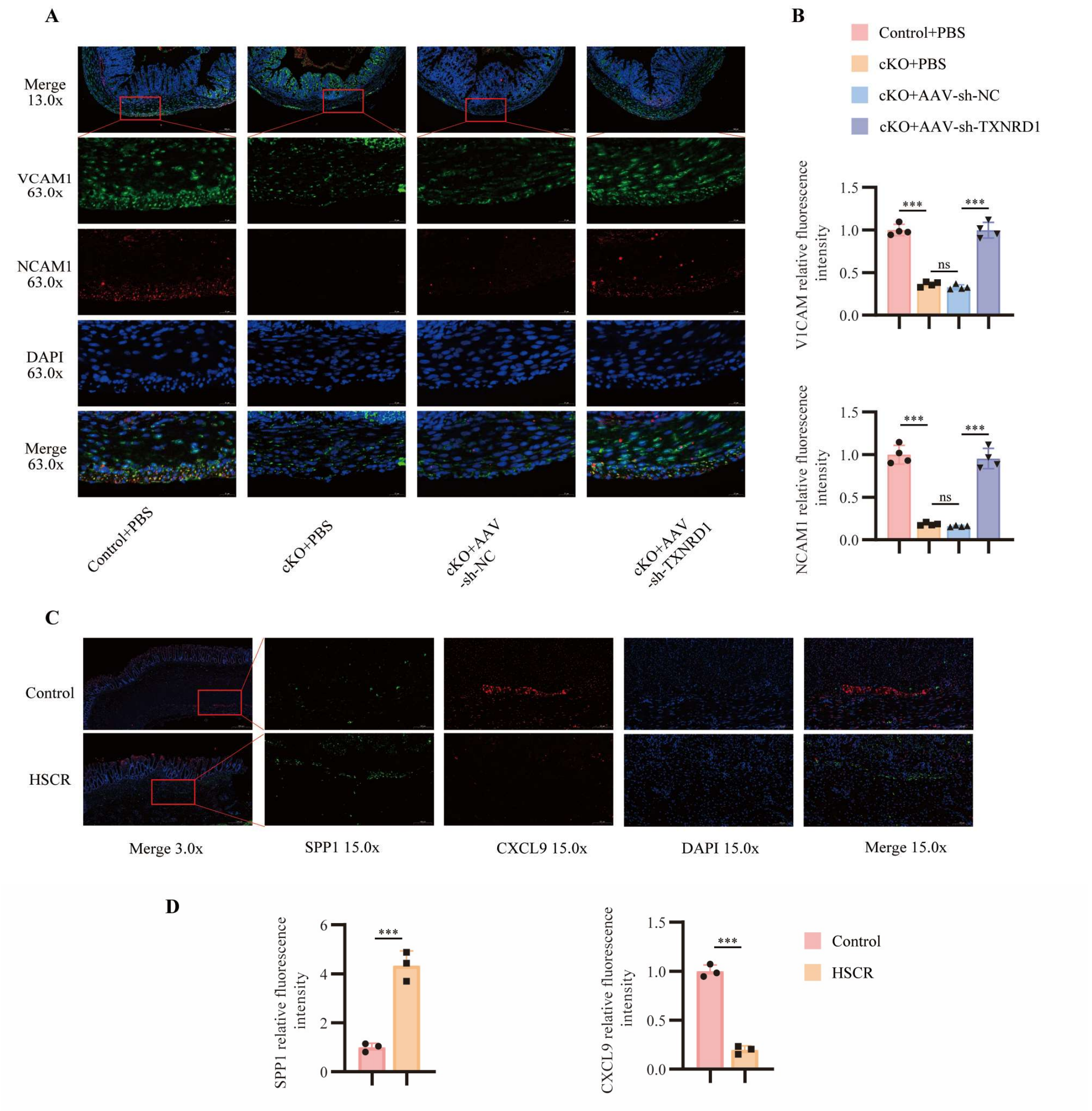

Figure S5 TXNRD1 knockdown restores VCAM1 and NCAM1 expression in *Ednrb* cKO mice, whereas human HSCR tissues exhibit SPP1-high/CXCL9-low remodeling.

A-B: Representative IF images of VCAM1 and NCAM1 in intestinal tissues from the four mouse groups described in Figure 6 (A) and quantification of relative VCAM1 and NCAM1 fluorescence intensity (B);

C-D: Representative IF images of SPP1 and CXCL9 in paired proximal dilated and stenotic colonic tissues from children with HSCR (C) and quantification of relative

fluorescence intensity (D).

For (A) and (B), n=4 mice per group; each dot represents one mouse. One-way ANOVA followed by Tukey's multiple-comparison test was used. For (C) and (D), n=3 paired patient samples; each dot represents one patient, and paired two-tailed Student's t tests were used. Quantitative data are presented as mean ± SD with individual data points. ***$P < 0.001$; ns, not significant. Micrographs are representative of four mice per group in (A) and three paired patient samples in (C). Scale bars, 100 μm in the overview images and 20 μm in the magnified images in (A); 500 μm in the overview images and 100 μm in the magnified images in (C).

Table S1. Primer sequences used for RT-qPCR and genotyping. Related to STAR Methods.

Gene	Forward primer (5'-3')	Reverse primer (5'-3')
TXNRD1(*Homo sapiens*)	TGTTCCTTATTTTGTGCTTGA	CCGCCTATCTTTCTCTGTTT
Txnrd1 (*Mus musculus*)	CTGGCGGTAGGAAGAGATT	TAAGGCACATTGGTCTGCT
Gapdh (*Homo sapiens*)	TCAAGAAGGTGGTGAAGCAG	AAGGTGGAGGAGTGGGTGT
GAPDH (*Mus musculus*)	GAGCGAGACCCCACTAACA	AGATGATGACCCTTTTGGCT
Wnt1-Cre	CAGCGCCGCAACTATAAGAG	CATCGACCGGTAATGCAG
	CAAATGTTGCTTGTCTGGTG	GTCAGTCGAGTGCACAGTTT
Ednrb-Flox	AGGGGGTCTAGAGGATGACC	GCCTAAAGGGACCTGAGGCA

Data S1. Original Western Blot images

Fig.2 D Uncropped Western blot images

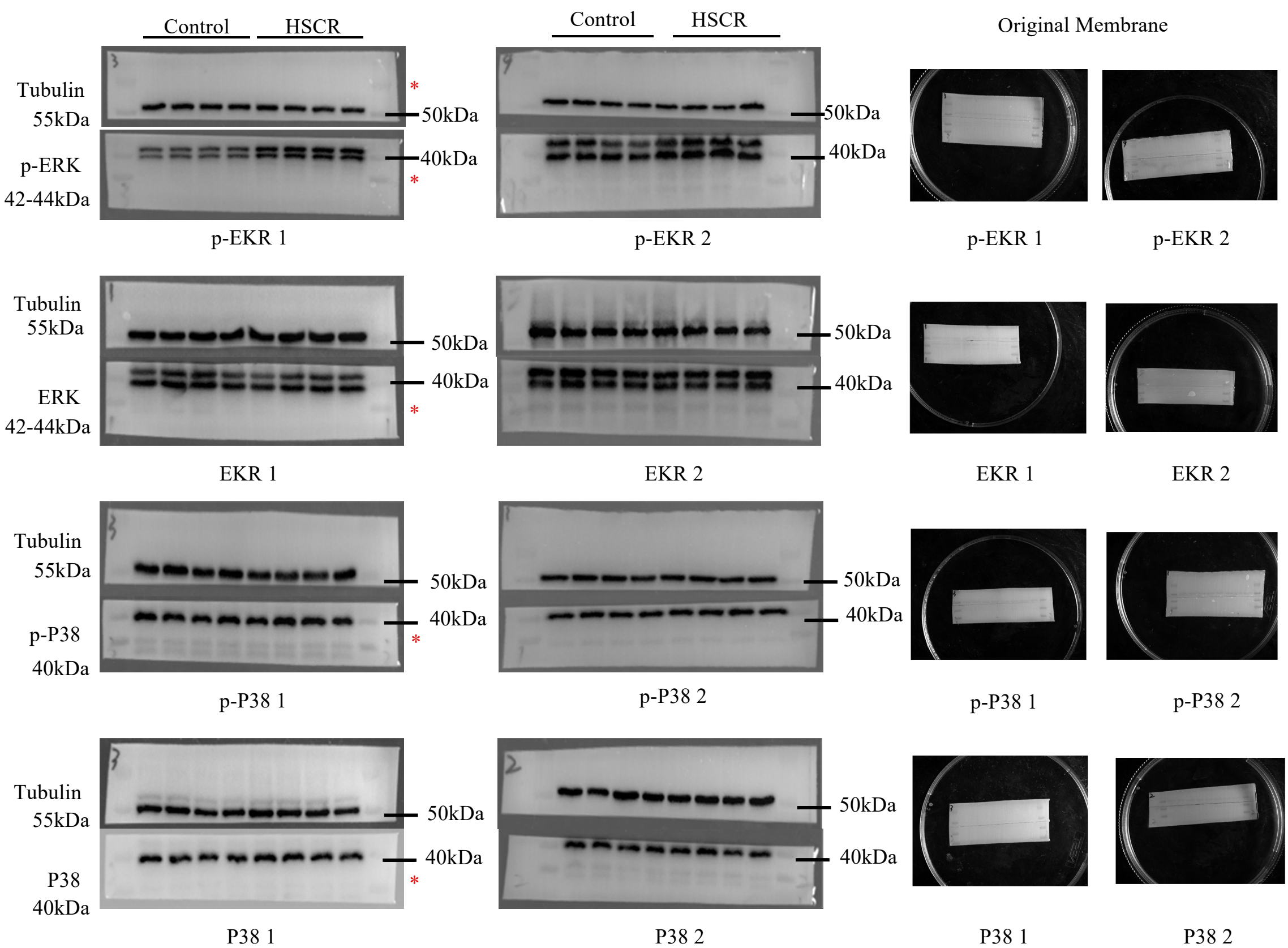

All bands were derived from the same original membrane. For clarity, different molecular-weight regions were cropped and assembled together. The relative positions of lanes were preserved, and no data manipulation was performed. * indicates the bands used in Fig.2 D.

Fig.2 E Uncropped Western blot images

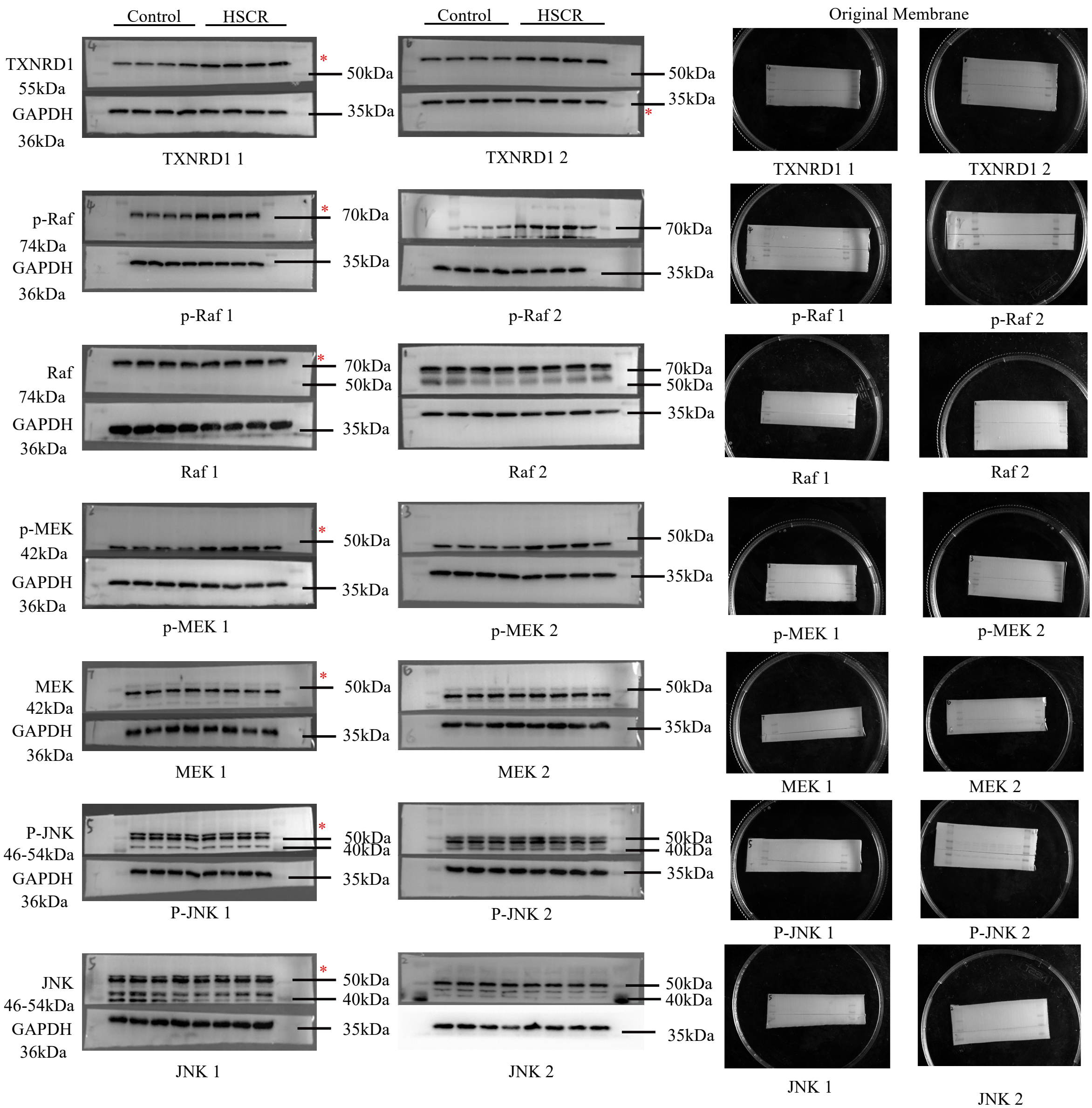

All bands were derived from the same original membrane. For clarity, different molecular-weight regions were cropped and assembled together. The relative positions of lanes were preserved, and no data manipulation was performed. * indicates the bands used in Fig.2 E.

Fig.3 B Uncropped Western blot images

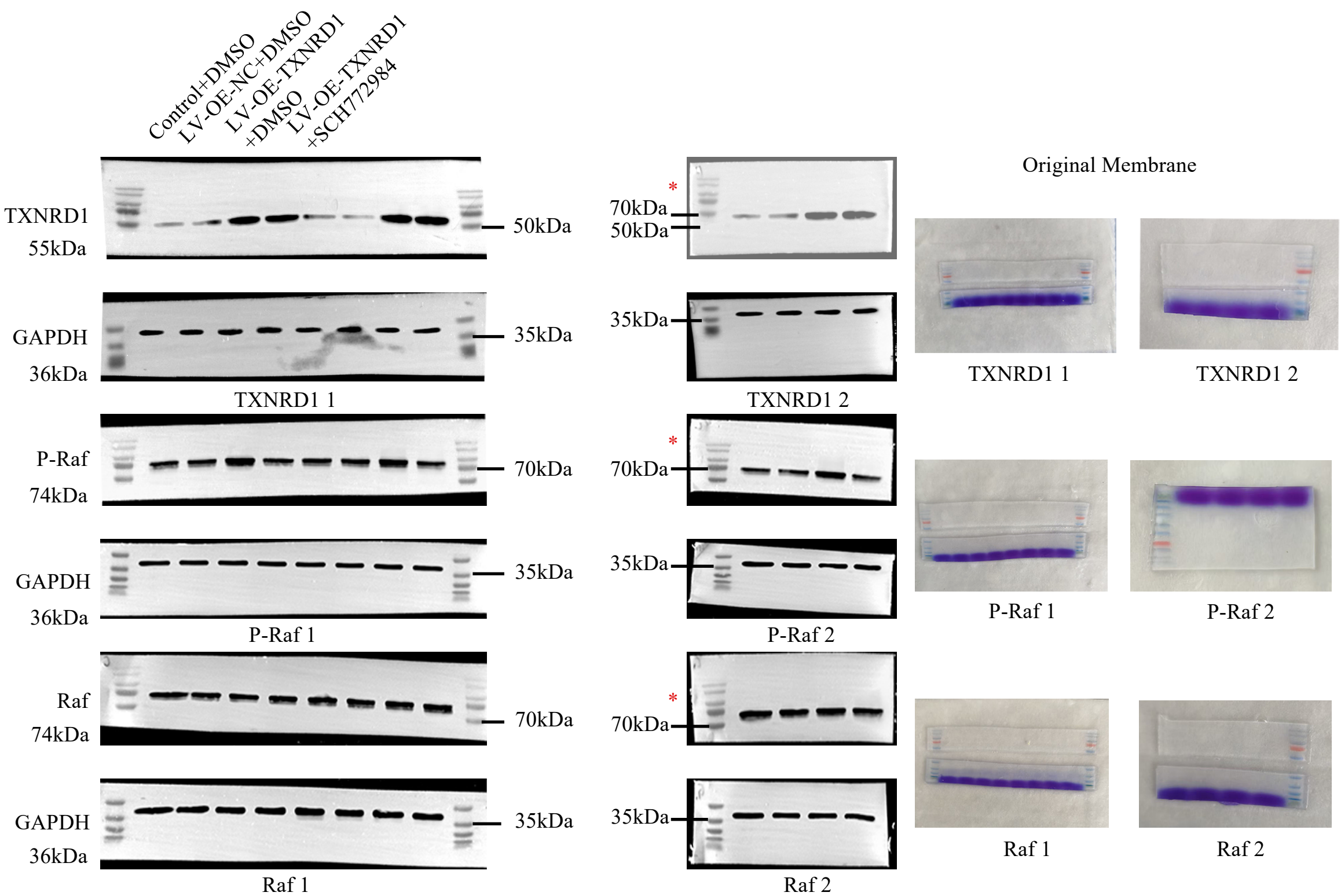

All bands were derived from the same original membrane. For clarity, different molecular-weight regions were cropped and assembled together. The relative positions of lanes were prserved,and no data manipulation was performed.* indicates the bands used in Fig.3 B.

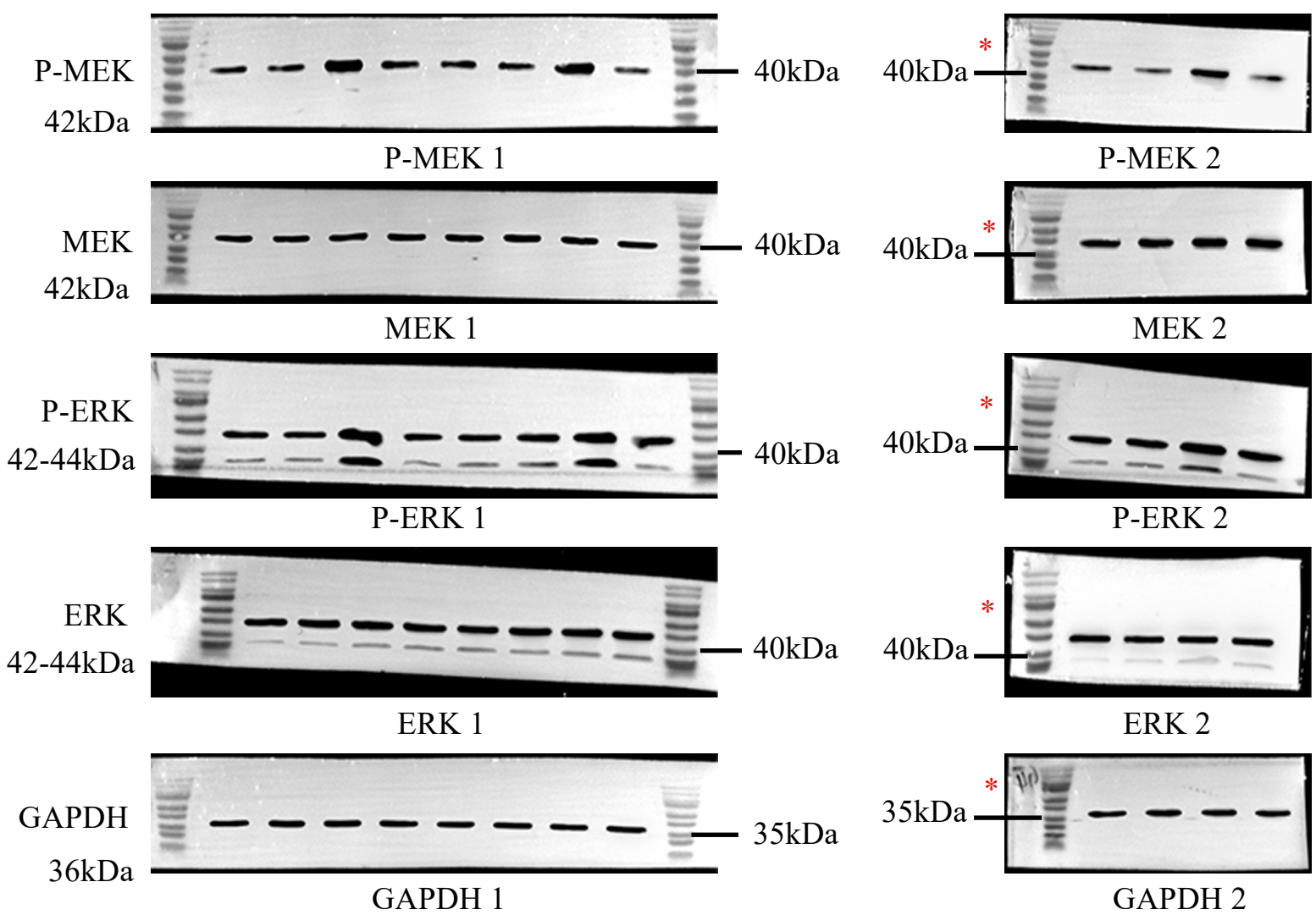

For Western blot analyses of p-MEK, MEK, p-ERK and ERK, 12 parallel SDS–PAGE gels were run simultaneously using the same batch of protein lysates with identical loading order and equal protein amounts. 10 membranes were probed for the indicated target proteins, and 2 parallel membranes were probed for GAPDH as the loading control. Therefore, the same GAPDH blot is shown as the internal control for these targets.* indicates the bands used in Fig.3 B.

Fig.4 A Uncropped Western blot images

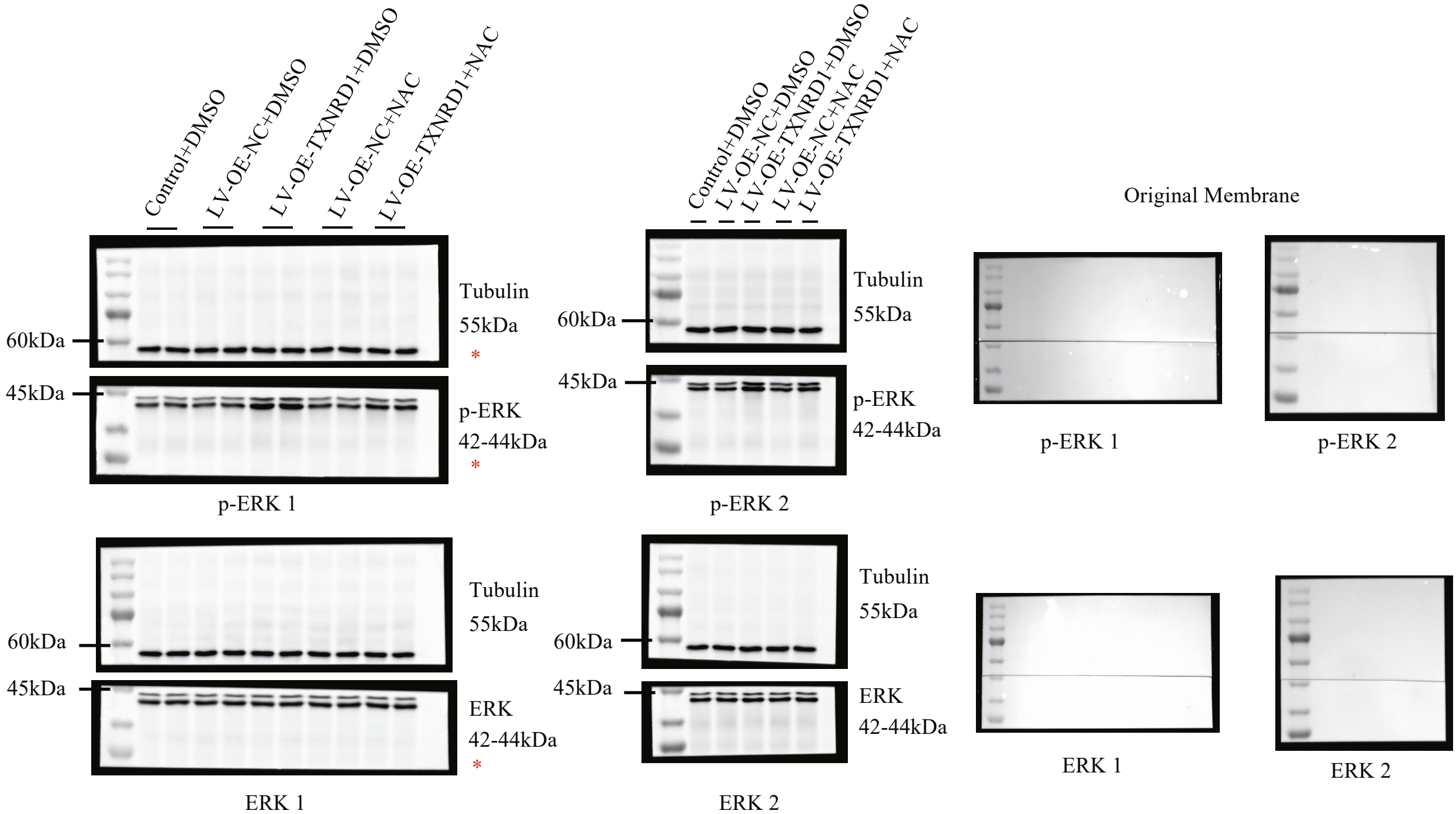

All bands were derived from the same original membrane. For clarity, different molecular-weight regions were cropped and assembled together. The relative positions of lanes were preserved, and no data manipulation was performed.* indicates the bands used in Fig.4 A.

Fig.5 D Uncropped Western blot images

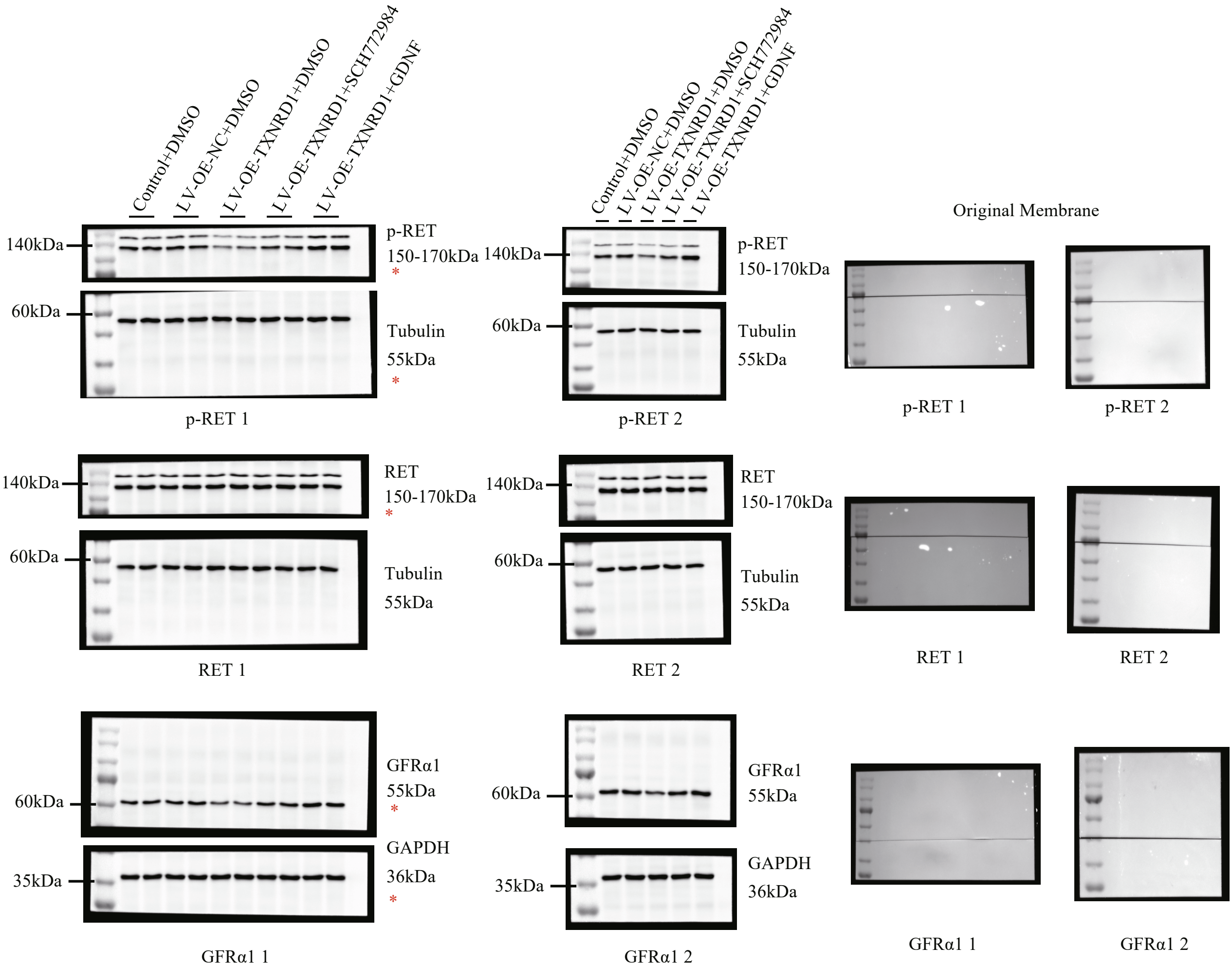

All bands were derived from the same original membrane. For clarity, different molecular-weight regions were cropped and assembled together. The relative positions of lanes were preserved, and no data manipulation was performed. * indicates the bands used in Fig.5 D.

Fig.6A Uncropped Western blot images

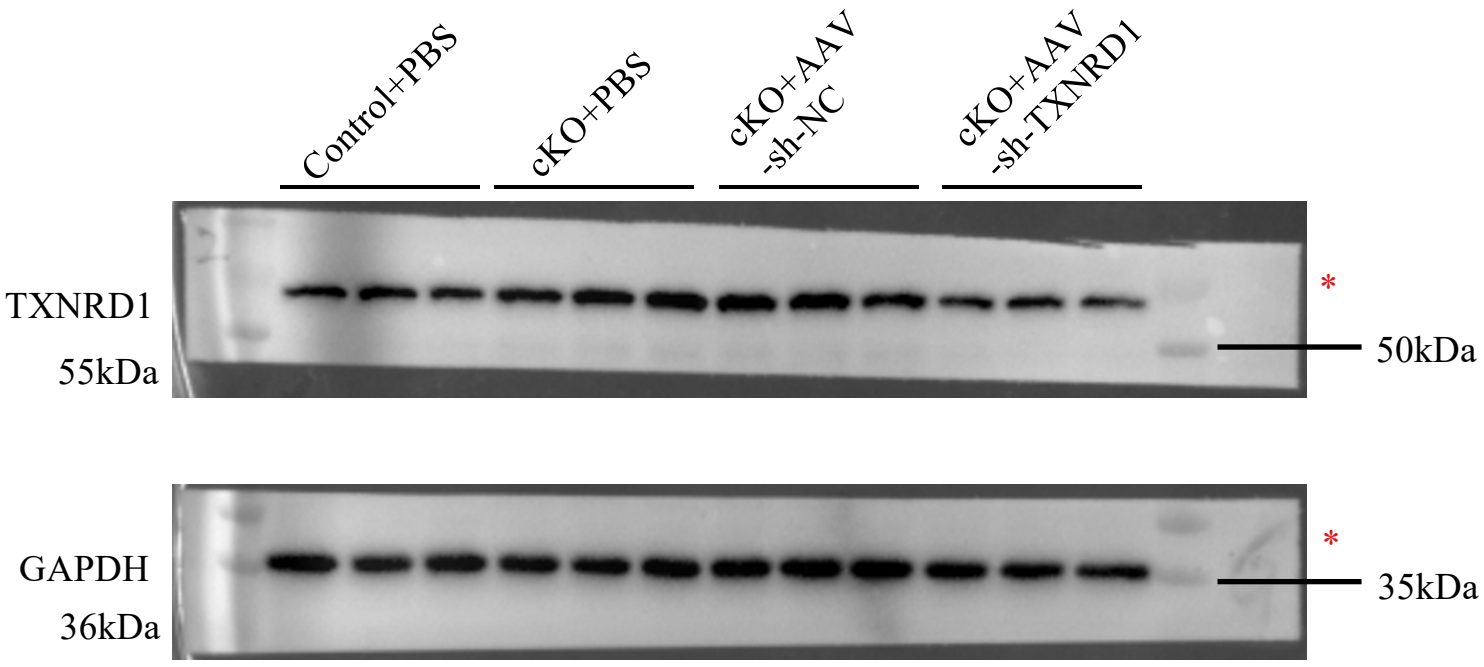

Original Membrane

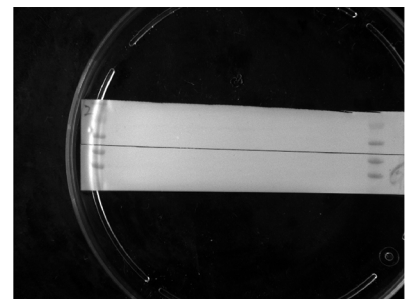

All bands were derived from the same original membrane. For clarity, different molecular-weight regions were cropped and assembled together. The relative positions of lanes were preserved, and no data manipulation was performed.* indicates the bands used in Fig.6 A.

Fig.S3 G Uncropped Western blot images

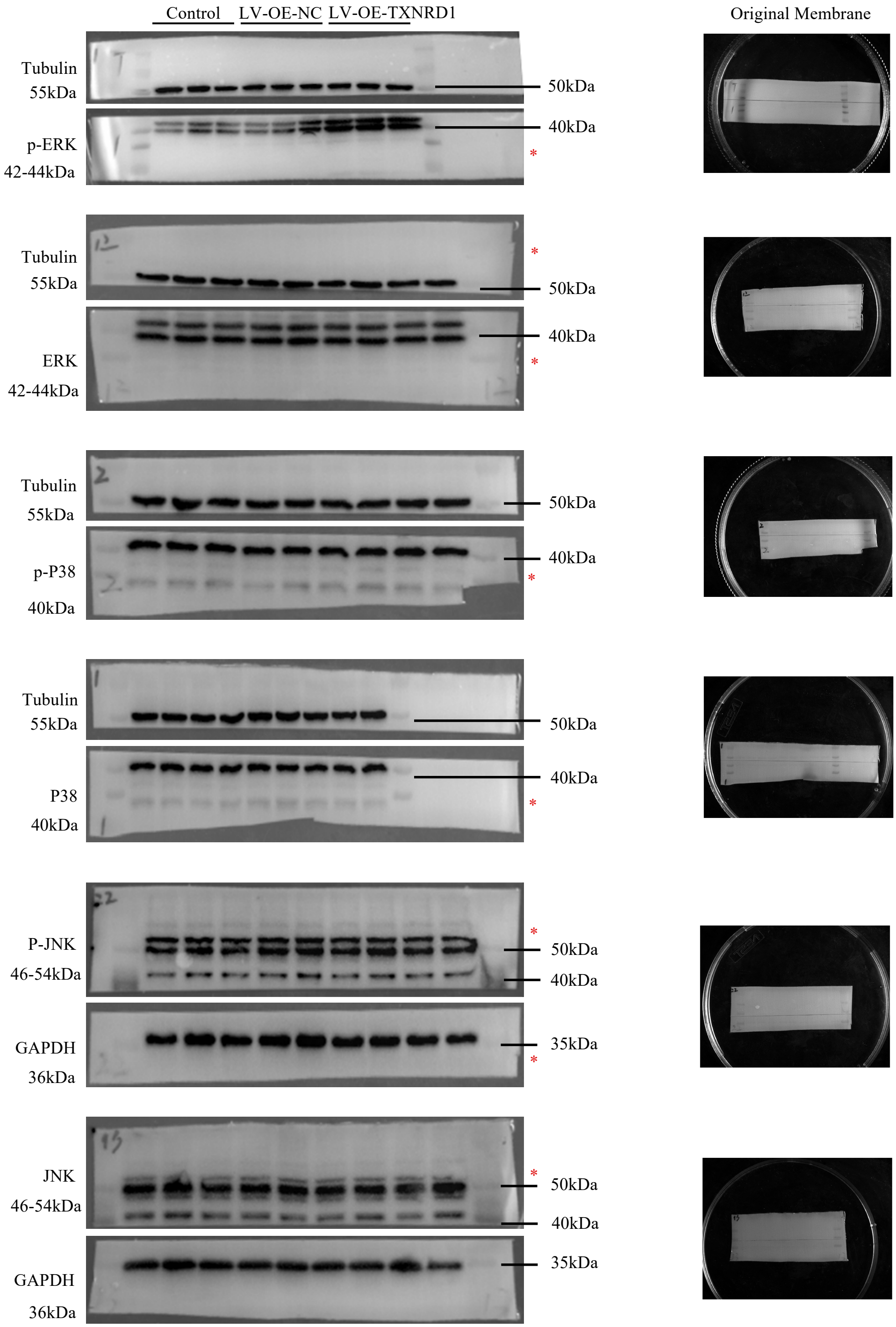

All bands were derived from the same original membrane. For clarity, different molecular-weight regions were cropped and assembled together. The relative positions of lanes were preserved, and no data manipulation was performed.* indicates the bands used in Fig.S3 G.
